# Liquid-liquid phase separation-driven molecular subtyping and prognostic modeling in colorectal cancer

**DOI:** 10.3389/fimmu.2025.1741979

**Published:** 2026-01-09

**Authors:** Hui Guan, Chengzi Tian, Jiawen Lin, Lihuan Zhang, Run Shi, Wenjing Wang, Shuping Li, Yuan Sui, Yanwen Lu, Tianjiao Cui, Duo Chen

**Affiliations:** 1 Department of Radiation Oncology, The First Affiliated Hospital of Zhengzhou University, Zhengzhou, Henan, China; 2 Department of Gynecology, The First People’s Hospital of Yunnan Province, The Affiliated Hospital of Kunming University of Science and Technology, Kunming, Yunnan, China; 3 Department of nephrology, Center of kidney and urology, the seventh affiliated hospital, Sun Yat-sen university, Shenzhen, Guangdong, China; 4 Center for Translational Medicine, The First Affiliated Hospital of Zhengzhou University, Zhengzhou, Henan, China; 5 Department of Oncology, The First Affiliated Hospital of Nanjing Medical University, Nanjing, Jiangsu, China; 6 Department of Biochemistry, SUSTech Homeostatic Medicine Institute, School of Medicine, Southern University of Science and Technology, Shenzhen, Guangdong, China; 7 Department of Physiology, University of Oklahoma Health Sciences Center, Oklahoma City, OK, United States; 8 Molecular and Cellular Biology Laboratory, The Salk Institute for Biological Studies, La Jolla, CA, United States; 9 Department of Urology, The First Hospital Affiliated of Zhengzhou University, Henan, China; 10 Department of Endocrinology and Metabolism, The First Affiliated Hospital of Zhengzhou University, Zhengzhou, Henan, China

**Keywords:** colorectal cancer, liquid-liquid phase separation, prognosis, spatial transcriptomics, tumor immune microenvironment

## Abstract

**Background:**

Liquid–liquid phase separation (LLPS) orchestrates the spatiotemporal organization of biomolecular condensates and regulates numerous biological processes. However, the extent to which dysregulated LLPS facilitates the progression of colorectal cancer (CRC) has not been elucidated. Elucidating how LLPS influences CRC possibly offers valuable insights into diagnosis and therapeutic intervention.

**Methods:**

Differentially expressed genes (DEGs) were identified from 566 CRC samples and 19 normal controls in the GSE39582 dataset. LLPS-linked genes were collected from the DrLLPS database. Prognostically significant genes were identified via univariate Cox regression, least absolute shrinkage and selection operator regression, and stepwise akaike information criterion algorithm. The risk score was derived utilizing the LLPS-linked gene signature. Patient characteristics were evaluated concerning the computed risk scores. The biological and clinical distinctions across high-risk and low-risk cohorts were further investigated, leveraging the COAD, READ, and GSE17536 validation cohorts. The expression and spatial distribution of the five prognostic genes were examined via the GSE166555 dataset and spatial transcriptomics analysis. The hydroxyacyl-coenzyme A dehydrogenase *(HADH)* expression-related enrichment pathways were further analysed via weighted gene coexpression network analysis combined with Metascape. The expression and biological functions of *HADH* were verified *in vitro*.

**Results:**

A total of 430 LLPS-related DEGs were identified, from which five prognostic genes were selected to construct the LLPS-associated risk signature. Marked differences in gene expression profiles, overall prognosis, clinicopathological attributes, somatic mutations, signaling pathway activity, tumor microenvironment composition, and drug sensitivity were noted across the high-risk and low-risk populations. Furthermore, the expression of the five prognostic genes and biological functions of *HADH* were validated through *in vitro* experiments.

**Conclusions:**

An LLPS-related prognostic model was created, enabling the stratification of the CRC population according to risk and informing individualized therapeutic strategies.

## Introduction

Liquid–liquid phase separation (LLPS) refers to the process by which biomolecules such as proteins and nucleic acids form liquid membraneless organelles (MLOs) through transient, low-affinity, multivalent weak interactions (1, 2). For example, nuclei, nuclear speckles, paraspeckles, Cajal vesicles in the nucleus, and stress granules and processors in the cytoplasm are all formed through LLPS (3, 4). LLPS-related biomolecules are functionally classified into scaffolds, clients, and regulators (5). Scaffolds are drivers of LLPS and are indispensable for MLOs, which can be formed by MLOs alone or with other molecules (6). Clients are recruited and regulated by scaffolds and do not undergo LLPS by themselves, forming MLOs with scaffolds only under certain circumstances (7). Regulators typically do not constitute the MLOs themselves but include other molecules, such as proteins, RNA, or ATP, that modulate the formation or stability of MLOs (8).

MLOs participate in many physiological and signaling pathways for heterochromatin formation, DNA damage repair, RNA metabolism, and autophagy through LLPS (9, 10). For instance, the MRN complex binds to DNA double-strand breaks (DSBs), and the MRN-interacting protein undergoes LLPS, which induces autophosphorylation of ataxia telangiectasia mutated protein, activates DNA damage response signaling, and promotes homologous recombination-mediated DSB repair (11). RAP80 undergoes LLPS at DSB sites, facilitating the rapid formation of Lys63-linked polyubiquitin chains, which in turn enhance RAP80 phase separation, recruit BRCA1, and contribute to DNA repair (12).

Dysregulated LLPS is crucial in malignant tumors (13, 14). For example, in triple-negative breast cancer cells, the disordered N-terminal domain and phosphorylated residues of histone deacetylase 6 promote aberrant LLPS, resulting in disorganized chromatin structure and uncontrolled cell proliferation (15). Mechanistically, DAZAP1, an RNA-binding protein, accumulates in the nucleus through LLPS, modulates pre-mRNA alternative splicing, enhances the expression of cytochrome-c oxidase 16, and promotes mitochondrial energy metabolism and invasion in oral squamous carcinoma (16). ARID1A, a chromatin remodeling factor, forms MLOs via prion-like domain-mediated LLPS, localizes to the EWS/FLT1 enhancer, and facilitates the formation of functional chromatin remodeling centers at oncogenic target sites, inducing long-range chromatin remodeling and driving proliferation and invasion in Ewing sarcoma (17).

Colorectal cancer (CRC) is a critical global health issue. In 2022, among 20 million new cases and 9.7 million deaths, CRC was the third most prevalent (1,926,118 cases; 9.6%) and second deadliest (903,859 deaths; 9.3%) (18). Its risk factors include obesity, smoking, and poor dietary patterns. The hallmarks of CRC pathogenesis include genomic instability, epigenetic dysregulation, and aberrant gene expression (19). Despite progress in systemic therapies such as surgery, radiotherapy, chemotherapy, and immunotherapy, a marked proportion of patients remain at risk for recurrence and metastasis, resulting in poor clinical outcomes (20). Therefore, unravelling pathogenic mechanisms and finding novel treatment targets are imperative to improve customized treatments and improve the prognosis of CRC patients.

LLPS is involved in the initiation, progression, and prognosis of CRC. For example, MEX3A in the cytoplasm of CRC cells undergoes LLPS, interacts with circMPP6, and the MEX3A/circMPP6 complex recruits processing bodies, enhances the degradation of PDE5A mRNA, which facilitates proliferation and invasion, and suppresses CRC cell autophagy, thus resulting in poor survival outcomes in CRC patients (21). In another example, oxaliplatin treatment alters nucleolar LLPS dynamics in CRC cells, impairing rRNA transcription and ribosomal function, triggering cell cycle arrest, and ultimately inducing cell death (22). However, many LLPS-associated molecules remain uncharacterized in the context of CRC, and their functional significance warrants further investigation.

Given these considerations, the exploration of LLPS-associated molecular mechanisms may provide critical insights into CRC pathogenesis and prospective therapeutic targets. LLPS-related differentially expressed genes (DEGs) across malignant and healthy tissues were identified. A prognostic signature was subsequently constructed, which demonstrated robust predictive performance. Furthermore, distinct clinical outcomes, biological functions, genomic alterations, features in tumor microenvironment (TME), and cancer stemness profiles were noted across high- and low-risk CRC subtypes. *In vitro* experiments verified that hydroxyacyl-coenzyme A dehydrogenase (*HADH*) significantly affected the proliferation, apoptosis and migration of CRC cells. These findings can improve prognostic evaluation and inform personalized therapeutic strategies for CRC patients.

## Methods

### Overall flowchart

**Figure 1:**
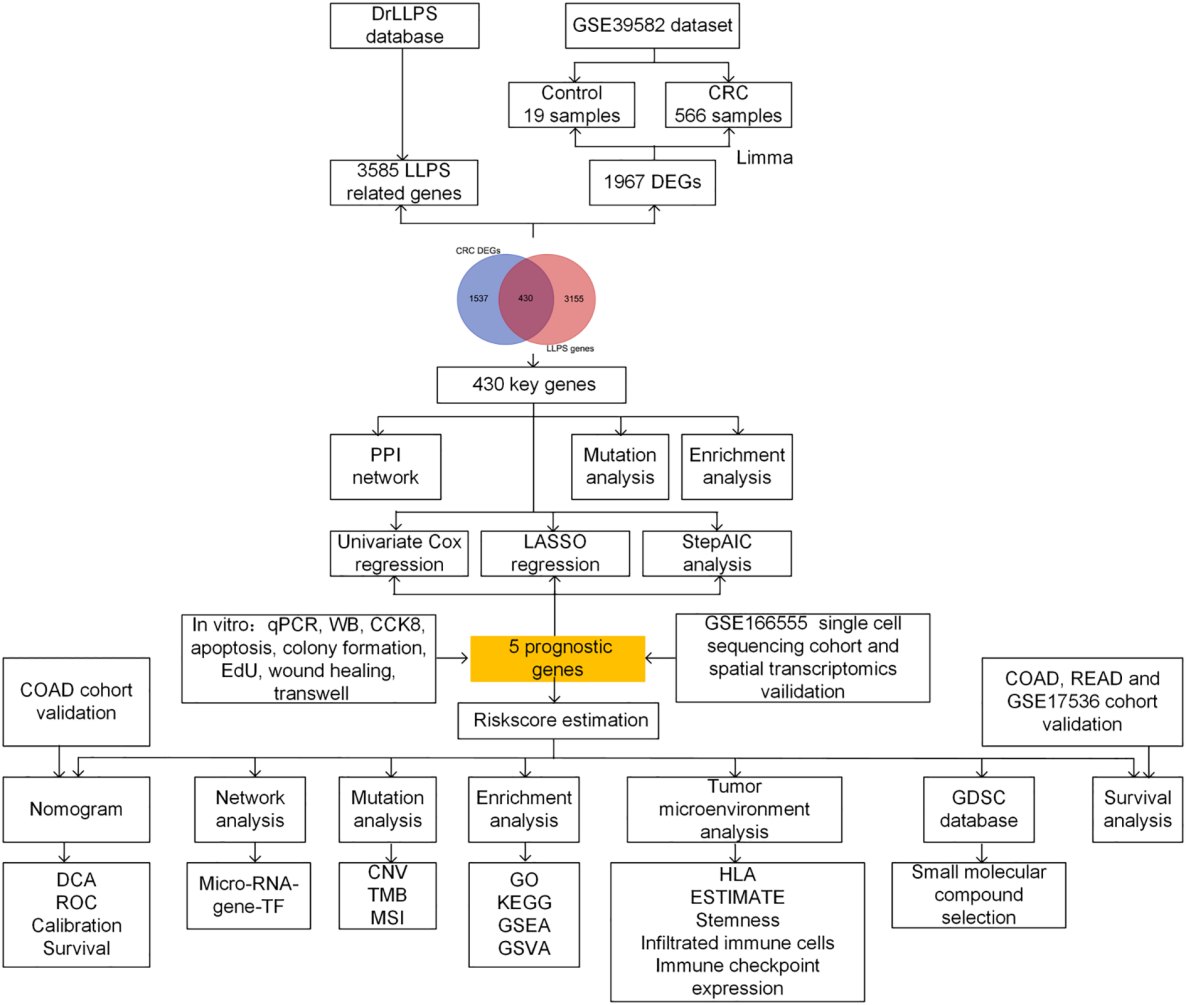
Flow chart of the study design.

The workflow is provided in **Figure 1**. Initially, DEGs were identified from the GSE39582 dataset. Genes overlapping with the LLPS-related gene set were considered core genes linked to CRC. The hub genes were subsequently screened via univariate Cox and least absolute shrinkage and selection operator (LASSO) regression, as well as the stepwise Akaike information criterion (stepAIC) algorithm. They were utilized to create a prognostic signature, with sample-specific risk scores derived accordingly. Differences in the TME, drug sensitivity, gene mutations, signalling pathways, and clinical prognosis were subsequently compared across high- and low-risk populations. The prognostic models were verified via the GSE17536, colon adenocarcinoma (COAD) and rectal adenocarcinoma (READ) datasets. The GSE166555 single-cell dataset and spatial transcriptome data were leveraged to assess prognostic gene expression and distribution. Finally, the expression and function of the hub genes were verified *in vitro*.

### Data collection

Normalized expression matrices containing RNA data from CRC patients were downloaded from the GEO database via the “GEOquery” package, with a particular focus on the GSE39582 and GSE17536 datasets developed on the GPL570 platform. Separate gene expression datasets for COAD and READ were also acquired from the TCGA database via the UCSC Xena interface (https://xena.ucsc.edu/) (23). Genes with missing expression values were removed, and only protein-coding genes with protein products were retained. Only genes present in both the annotation database and the expression matrix were preserved to ensure biological relevance in subsequent analyses. For cases where multiple probes corresponded to the same gene, the probe with the highest expression level was selected to represent that gene.

After removing samples lacking prognostic information, we subsequently analyzed 566 malignant and 19 control samples from the GSE39582 dataset, 177 tumor samples from GSE17536, 430 tumor samples from the COAD dataset, and 154 tumor samples from the READ dataset (24, 25). Details of all datasets were provided in **Supplementary Table S1**. Leveraging 10x Genomics single-cell 3’ library technology, scRNA-seq data from twelve CRC patients in the GSE166555 dataset were retrieved from the GEO database. Spatial transcriptomic analysis of three CRC patients was performed via the Sparkle website (https://grswsci.top/). Information on genes related to LLPS was acquired from data resource of liquid–liquid phase separation database (DrLLPS, http://llps.biocuckoo.cn/) (26). A total of 3,585 genes specific to humans (85 scaffolds, 355 regulators, and 3,145 clients) were isolated for downstream investigation, details were listed in **Supplementary Table S2** (27). All LLPS-related genes were assigned equal weight.

### DEG screening and functional analysis

DEGs across tumor and control tissues were identified via the “limma” package in the GSE39582 dataset, and the results were displayed as volcano plots. Statistically significant genes were defined as those with a |log2-fold change| > 1 and an adjusted P ≤ 0.05. The overlap between DEGs and LLPS-related genes was illustrated via Venn diagrams. The expression of intersecting genes was presented in a heatmap generated with the “pheatmap” package. To elucidate their biological functions, Gene Ontology (GO) and Kyoto Encyclopedia of Genes and Genomes (KEGG) enrichment analyses were performed via the “clusterProfiler” package. Multiple testing correction was performed using the Benjamini–Hochberg method, and the top 15 enriched pathways were selected for visualization bar plots. Additionally, a protein–protein interaction (PPI) network was constructed via STRING (https://cn.string-db.org/) to assess possible interactions among the shared genes.

### Construction of an LLPS-linked gene signature

A three-step approach was employed to develop a robust LLPS-associated gene signature for prognostic evaluation utilizing the GSE39582 dataset. First, univariate Cox regression analysis identified DEGs linked to overall survival (OS). Next, the LASSO regression algorithm, implemented via the “glmnet” package, was applied to further screen the variables. The stepAIC from “MASS” subsequently refined the selection to retain the most prognostically significant genes. Finally, these key LLPS-related genes were integrated into a multivariate Cox proportional hazards model. The gene signature was derived via the following methods:


$$\text{Risk score}=\sum_{i=1}^{N}\left(Expi\times Coei\right)$$

In the model, “Expi” denotes the expression of a gene, whereas “Coei” indicates its corresponding coefficient. With these variables, every patient was assigned a risk score according to the LLPS-associated gene signature. The participants were then divided into high- and low-risk cohorts via the median risk score as the cut-off. The “survival” package facilitated Kaplan–Meier survival analysis and log-rank testing for OS comparison across groups. The “ggrisk” package was employed to graphically display the risk score distribution, survival duration, and mortality status. For external validation, the GSE17536, COAD, and READ datasets were analysed to evaluate the broader applicability of this LLPS-based signature.

### Nomogram model development

The prognostic independence of clinical parameters (age, chemotherapy, clinical stage, T, N, and M classifications, and risk score) was estimated in the GSE39582 dataset via univariate and multivariate Cox regression analyses. A prognostic nomogram was subsequently generated utilizing the aforementioned significant risk factors with “rms” and “survival” package. To determine the nomogram’s predictive precision, researchers have employed the area under the receiver operating characteristic (ROC) curve (AUC), Kaplan–Meier survival analysis, calibration curves, and decision curve analysis (DCA). The COAD cohort data provided external validation of the model’s performance.

### TME evaluation

Additionally, the expression profiles of immune checkpoint-linked molecules and human leukocyte antigen (HLA) genes were examined. Estimation of stromal and immune cells in malignant tumour tissues using expression data (ESTIMATE) method based on single-sample gene set enrichment analysis (ssGSEA) algorithm was applied to infer tumor purity, stromal, immune, and ESTIMATE scores (combining stromal and immune components) via the “estimate” package (28). The tumor immune dysfunction and exclusion (TIDE, https://tide.nki.nl/) score was employed to predict the response to immunotherapy between the two groups. The CIBERSORT method based on linear support vector regression was conducted to assess the composition of tumor-infiltrating immune cells via the “CIBERSORT” package (29). Furthermore, disparities in immune cell infiltration were evaluated across high-risk and low-risk populations. The relationships among immune cells and their potential correlations with hub gene expression were revealed via the “corrplot” package.

### Stemness signature analysis

StemChecker (http://stemchecker.sysbiolab.eu/) was utilized to identify and examine stemness-associated signatures within the relevant gene sets (30). A collection of 26 stemness gene sets was first obtained from StemChecker, incorporating diverse data types such as transcriptional profiles, RNAi screening results, manually compiled literature, computationally inferred transcription factor (TF)-regulated genes, and Nanog-linked signatures. The stemness score was evaluated based on ssGSEA by calculating the similarity between tumor cells and predefined stem cell gene signatures.

### Functional enrichment analysis

DEGs across two risk cohorts in the GSE39582 dataset were identified and visualized via a volcano plot. The possible biological functions of these genes were examined through GO enrichment analysis. The enriched pathways and biological processes were further integrated via the Metascape database (https://Metascape.org/gp/index.html) (31). GSEA based on KEGG pathways was performed with the “clusterProfiler” package to identify differentially enriched pathways between two risk groups, applying the thresholds of |normalized enrichment score (NES)| > 1, nominal p < 0.05, and q < 0.25 (32). Additionally, gene set variation analysis (GSVA) was carried out via “GSVA” package to derive enrichment scores of predefined gene sets across samples, with the threshold of adjusted P < 0.05.

### Comprehensive network analysis

NetworkAnalyst (http://www.networkanalyst.ca) is an application that maps genes of interest to related databases and performs network analysis online (33). Five LLPS-related genes were uploaded to the NetworkAnalyst website, and their links to TFs and miRNAs were analysed. Regulatory interaction data, which were carefully extracted from published studies, were acquired through the RegNetwork repository. TF–gene–miRNA interaction networks were visualized via Cytoscape 3.10.2.

### Somatic variant analysis

Somatic variant data for CRC were downloaded from the UCSC Xena platform, and mutation patterns in 430 DEGs were analyzed using the “maftools” package (34). Additionally, copy number variation (CNV) data encompassing gene amplifications and deletions were retrieved from the UCSC Xena platform for COAD and READ cohorts to analyze mutation patterns of the 430 DEGs.

### Single cell RNA sequencing analysis

The “Seurat”package facilitated the conversion of scRNA-seq analysis data of GSE166555 dataset into an analysable object. After normalization (NormalizeData), highly variable genes were selected (FindVariableFeatures) and data were scaled. Batch-corrected principal component analysis was conducted using these genes. Cell clusters were identified at a resolution of 0.8 and annotated against marker databases. Subsequently, cell clustering and annotation were performed, followed by dimensionality reduction and visualization using the Uniform Manifold Approximation and Projection (UMAP) method. The activity of 430 LLPS-related DEGs in each cell was subsequently quantified with the “AUCell” package and visualized with the “Seurat” package (LLPS enrichment score). The expression and distribution of the five prognostic genes across cell types were examined. Taking normal epithelial cells as the reference, the CNV level of tumor epithelial cells was evaluated via the “inferCNV” package. The cells were divided into high and low groups on the basis of the median value of the CNV score, and the expression levels of five prognostic genes in the two groups were evaluated.

### Spatial transcriptome analysis

Spatial transcriptomics enabled the spatial distribution and expression evaluation of the five prognostic genes in the tumor tissues of the CRC population. Initially, the cellular composition of each microregion was estimated via inverse convolution methods. Quality control procedures were implemented on the basis of gene expression counts, unique molecular identifiers, and the mitochondrial RNA content per cell. A signature score matrix was created by averaging the expression of the top 25 cell sorting-specific genes for each microregion. Enrichment score matrices were generated via the “Cottrazm” package. Microregions with enrichment scores equal to 1 were classified as malignant; others were considered nonmalignant. Differential expression of the five prognostic genes across subcohorts was examined via the Wilcoxon rank-sum test. Spearman correlation analyses between gene expression and cellular composition were presented as a heatmap generated with the “linkET” package.

### Screening of potential small molecule therapeutics for CRC

Data on tumor cell sensitivity to small-molecule compounds were retrieved from Genomics of Drug Sensitivity in Cancer 2 (GDSC2) database (https://osf.io/c6tfx/) (35). Drug sensitivity (represented as the IC50) was predicted for each sample in the GSE39582 cohort via the “oncoPredict” package. Spearman correlation coefficients were used to explore the relationships of the expression of the five prognostic genes with the IC50 values. Disparities in the IC50 across high- and low-risk cohorts were visualized as violin plots. The chemical structures of the compounds were obtained from ChemSpider (http://www.chemspider.com) and rendered via ChemDraw software (version 22.0.0.22).

### Biological functions of *HADH* genes analysed via weighted gene coexpression network analysis

We further compared the expression levels of the five prognostic genes and OS in the high-risk and low-risk groups. The results of the comprehensive analysis of the bulk RNA transcriptome, scRNA-seq, and spatial transcriptome suggested that *HADH* was downregulated in tumor tissues and was expressed mainly in tumor cells with low CNV, as was its favourable prognostic value. This gene was selected for further functional investigation. First, a scale-free coexpression gene network and average connectivity were constructed via the “WGCNA” package to optimize the soft threshold power. The modular trait was subsequently constructed to evaluate the associations between different module eigengenes and *HADH* expression. The module with the most significant positive correlation of module-*HADH* expression was screened. The module genes were imported into the Metascape website to explore the pathways relevant to *HADH* expression.

### Immunohistochemistry

Tissue microarrays comprising tumor specimens and paired adjacent nontumorous tissues from 80 individuals with CRC were obtained from AIFANG Biotech Co., Ltd. (AF-CocSur2201, China). The tissues were first dewaxed and rehydrated. The antigens were retrieved with EDTA buffer. The endogenous peroxidase activity of the sections was subsequently inhibited with hydrogen peroxide, and the sections were blocked with 3% BSA. The sections were incubated with an antibody that targets HADH (Proteintech, 19828-1-AP, China) at 4 °C overnight. After washing, a secondary antibody (Servicebio, GB23303, China) was applied for 1 hour. DAB was used to visualize the immunoreaction, and hematoxylin was used to contrast the cell nuclei. The stained samples were imaged and analysed under a microscope (KF-FL-020, KFBIO, China). For scoring, staining intensity was classified as 0 (no staining), 1 (light yellow), 2 (tan), or 3 (deep brown). The percentages of positively stained cells were 0 (0–5%), 1 (6–25%), 2 (26–50%), 3 (51–75%), or 4 (>75%). The ultimate immunoreactivity score (IRS) was obtained by multiplying the intensity and percentage. Tumor tissues were divided into high- and low-HADH expression cohorts according to the median IRS value. Clinical and pathological differences across cohorts were compared, and the results were visualized via the “ComplexHeatmap” package.

### Cell culture

Colorectal cancer cell lines (HCT116, RKO, HT29, HCT8, SW620, and KM12), immortalized NCM460 epithelial cells, and HEK-293T cells were obtained from OriCell (China). HT29 cells were cultured in McCoy’s 5A medium (G4541, Servicebio, China) supplemented with 10% fetal bovine serum (FBS, A5256701, Gibco, USA) and 1% penicillin/streptomycin (P/S, 15140122, Gibco, USA), and the other cells were cultured in DMEM (11965092, Gibco, USA) supplemented with FBS and P/S. All the cells were placed in a humid incubator (Thermo Fisher Scientific, USA) (37°C, 5% CO_2_).

### Lentiviral transfection

The HADH overexpression plasmid (HADH), control plasmid (NC), and helper plasmids (pSPAX2 and pMD2G) were purchased from Miao Ling Company (China). HEK-293T cells were transfected with plasmids containing polyethylenimine (40816ES01, Yeasen, China) and cultured for 72 h. The lentivirus-containing cell supernatant was subsequently collected. The KM12 and RKO cells were incubated with the lentiviral supernatant and 10 μg/mL polybrene (C0351, Beyotime, China) for 48 hours and then with puromycin (2 μg/mL; ST551, Beyotime, China). Successful overexpression was confirmed by real−time quantitative polymerase chain reaction (RT−qPCR) and Western blotting.

### RT−qPCR

Total cellular RNA was extracted with TRIzol reagent (15596026CN, Thermo Fisher Scientific, USA). cDNA was derived via reverse transcription according to the manufacturer’s guidelines for the Reverse Transcription Kit (R433, Vazyme, China). Fifteen pairs of tumor and matched paracancerous cDNA microarrays were procured from Outdo Biotech Company (MecDNA-HColA030CS03, China). qPCR was performed with a SYBR Green PCR Kit (Q712, Vazyme, China). The primer sequences were detailed in **Supplementary Table S3**. The relative expression of mRNA was computed via the 2^-ΔΔCt^ approach and standardized with β-actin as the internal control.

### Western blotting

The cells were lysed in RIPA buffer (CW2333S, CWBIO, China) supplemented with 1% protease inhibitor cocktail (CW2200S, CWBIO, China) for 30 minutes. Protein concentrations were determined via a BCA kit (23227, Thermo Fisher Scientific, USA). Proteins were transferred onto PVDF membranes (IPVH00010, Millipore, USA) after being separated via SDS–PAGE. After being blocked for an hour in 5% nonfat milk, the samples were incubated overnight at 4°C with antibodies targeting HADH or β-tubulin (1:5000, 80762-1-RR; Proteintech, China). The membranes were then incubated with HRP-conjugated secondary antibodies (GAR1007, MultiSciences, China) for 1 hour. Following washes, protein signals were detected via a chemiluminescence reagent (GK10008, GLPBIO, USA). Densitometric analysis of HADH expression relative to that of β-tubulin was performed via ImageJ.

### Cell viability assay

Briefly, 1,000 cells per well were seeded in 96-well plates. At 0, 24, 48, 72, and 96 h, the cells were treated with culture medium supplemented with 10% CCK-8 solution (GK10001, GLPBIO, USA) and incubated for 2 h at 37°C. The optical density at 450 nm was measured via a microplate reader (Synergy H1, BioTek, USA).

### Colony formation assay

Cells were seeded in plates with 6 wells with1,000 cells in each well and cultured with medium replacement every 72 h. After two weeks, the cells were rinsed with PBS, fixed with 4% paraformaldehyde (G1101, Servicebio, China) for 30 min, and stained with crystal violet reagent (C0121, Beyotime, China) for 30 min. Colonies were visualized via the Amersham ImageQuant 800 system (Cytiva, Japan), and quantification was performed via ImageJ.

### Cell proliferation assay

The cells were seeded in 6-well plates and incubated for 24 hours before they were incubated with an EdU solution (C0078S, Beyotime, China) for 4 hours. Following fixation in 4% paraformaldehyde, the cells were washed three times with PBS supplemented with 3% BSA and incubated with permeabilization solution (P0097, Beyotime, China) for 15 minutes. The samples were incubated with click additive solution for half an hour, stained with Hoechst for 10 minutes, and visualized via a microscope (BX53, Olympus, Japan). The ratio of EdU-positive cells was determined via ImageJ software. For each well, the percentage of EdU-positive cells was determined by counting approximately 300 cells per field across five randomly selected fields of view, and the mean value was calculated.

### Apoptosis assay

Apoptosis was assessed via an Annexin V/PI detection kit (HY-K1093, MedChemExpress, USA). The cells were cultured for 24 hours and harvested via trypsin digestion (C0207, Beyotime, China). Subsequent to washing with PBS, the cell suspensions in binding buffer were subjected to 15 minutes of incubation in the dark with both Annexin V and PI stains. Fluorescent signals were detected via flow cytometry (Canto, BD Biosciences, USA). Data analysis was enabled by FlowJo 10.8.1 (Tree Star, USA).

### Wound healing assay

The cells were seeded in 12-well plates until approximately 90% confluence was achieved. Mechanical wounds were created with a 10 μl pipette tip. After being washed, the cells were cultured in serum-free medium. The observation time points included immediately after wounding and 24 hours after wounding, and a microscope was used. The area of wound closure was quantified via ImageJ.

### Transwell assay

Transwell chambers (8 μm pore polyester membrane inserts; TCS020024, Jet, China) were either uncoated or precoated with 60 μl of matrix gel (082704T, Mogengel, Xiamen, China) to assess cell migration or invasion. Cells (25,000 KM12 or 100,000 RKO per well) suspended in serum-free medium were seeded into the upper chambers. The lower chambers were supplemented with 600 μl of medium containing 20% FBS as a chemoattractant. After 48 h, the cells remaining in the upper chambers were removed. The inserts were then fixed with paraformaldehyde and stained with crystal violet. The cells that had migrated or invaded were visualized and quantified via a microscope and ImageJ.

### Statistical analysis

All data originating from public databases were analysed via R 4.4.1. All experiments were performed in triplicate with three technical replicates, and independently repeated three or more times as biological replicates. The results are presented as the mean ± SEM of independent determinations. Normally distributed variables in the two cohorts were compared via unpaired t tests, whereas nonnormally distributed variables were explored via the Mann–Whitney test. The log-rank test was used to evaluate survival disparities through Kaplan–Meier survival curves. The false discovery rate (FDR) was controlled through Benjamini–Hochberg correction.

## Results

### LLPS-related DEGs were identified in the CRC cohort

**Figure 2:**
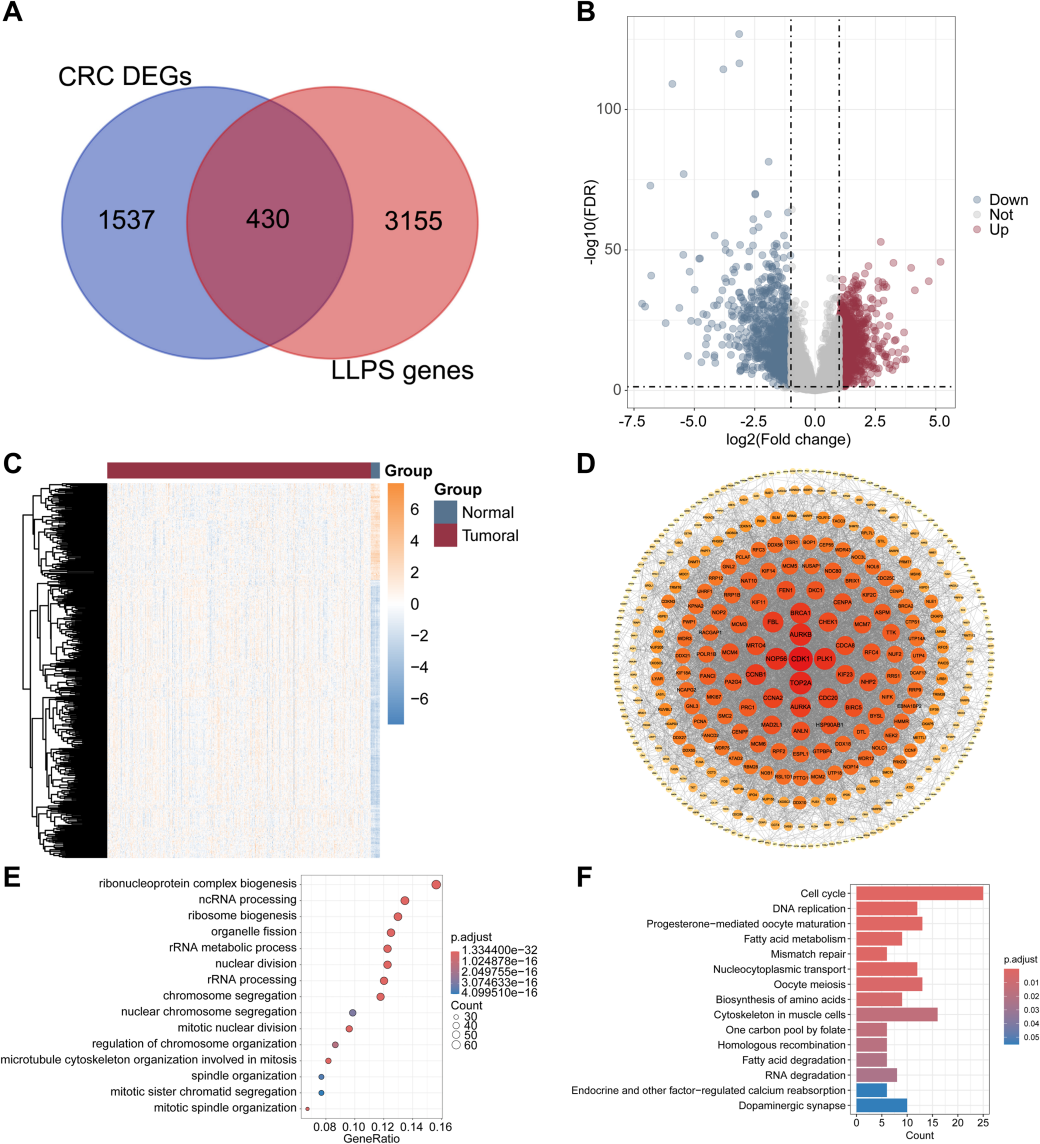
Identification of LLPS-related genes in CRC patients. **(A)** Venn diagram showing 430 LLPS-related DEGs between the tumor and normal cohorts. **(B)** Volcano plot representing 1967 DEGs in the GSE39582 dataset. **(C)** Heatmap illustrating the expression of the 430 LLPS-linked DEGs. **(D)** The PPI network elucidated the intricate relevance of 430 gene-associated proteins. **(E)** GO enrichment analyses of 430 LLPS-related DEGs. **(F)** KEGG enrichment analyses of 430 LLPS-related DEGs.

A total of 1,967 genes demonstrated significant differences in expression (1,087 with elevated expression and 880 with reduced expression) in tumor versus normal tissues within the GSE39582 dataset (**Figure 2A**). Among these DEGs, 430 DEGs related to LLPS were identified between tumor and normal tissues, representing distinct transcriptomic features (**Figure 2B**). The expression of these 430 LLPS-linked DEGs in the GSE39582 cohort was presented as a heatmap (**Figure 2C**). The complex interactions among the proteins encoded by the 430 DEGs were revealed via a protein–protein interaction (PPI) network (**Figure 2D**). The biological functions and regulatory mechanisms of LLPS-linked DEGs were elucidated via GO and KEGG enrichment analyses. The former revealed significant enrichment in ribonucleoprotein complex biogenesis, noncoding RNA processing, ribosome biogenesis, organelle fission, and ribosomal RNA metabolic processes (**Figure 2E**). The latter indicated enrichment in pathways related to the cell cycle, DNA replication, ribosome biogenesis in eukaryotes, progesterone-mediated oocyte maturation, and fatty acid metabolism (**Figure 2F**).

### The LLPS related gene signature was constructed to predict prognosis

**Figure 3:**
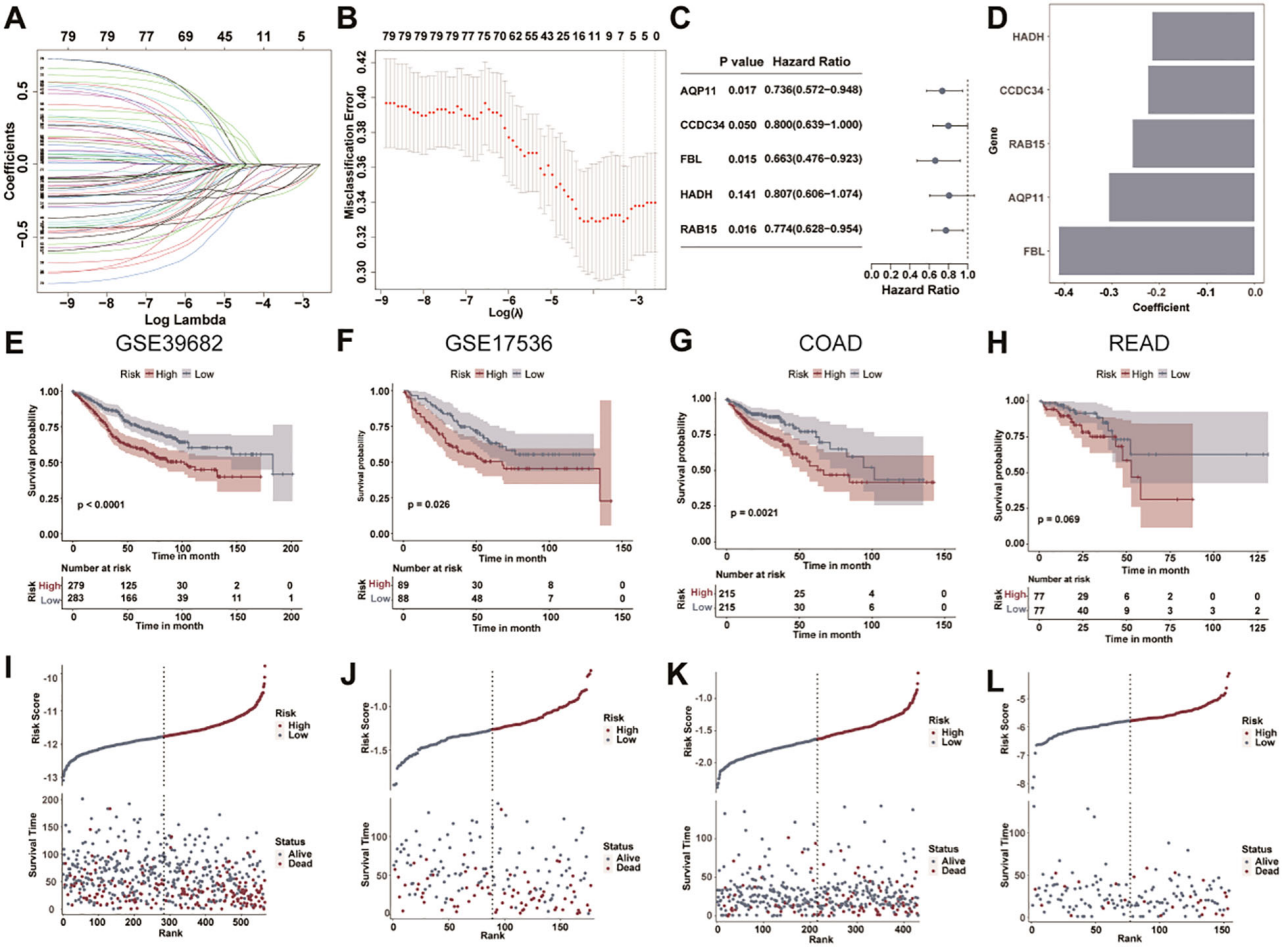
Construction of the LLPS-linked prognostic signature for CRC. The minimum **(A)** and lambda values **(B)** of the prognostic genes (n = 7) were selected via the LASSO algorithm. **(C)** The forest plot displays the prognostic model based on five LLPS-linked genes. **(D)** The histogram shows the five prognostic genes with coefficients. Kaplan‒Meier survival curves revealed different OS rates across high- and low-risk populations in the GSE39582 **(E)**, GSE17536 **(F)**, COAD **(G)**, and READ **(H)** cohorts. The distributions of risk scores and survival were shown for the GSE39582 **(I)**, GSE17536 **(J)**, COAD **(K)**, and READ **(L)** cohorts.

The prognostic model construction was conducted as follows: Starting from 430 DEGs, 79 genes were identified via univariate Cox regression. Subsequent LASSO regression narrowed the candidates to seven genes, and stepAIC optimization finalized a five-gene signature (**Figures 3A–C**). Multivariate Cox proportional hazards modeling generated coefficients for these genes, yielding the risk score formula: Risk score = (-0.3059) × aquaporin 11 (*AQP11*) + (-0.2237) × coiled-coil domain-containing protein 34 (*CCDC34*) + (-0.4115) × fibrinogen-like protein (*FBL*) + (-0.2149) × *HADH* + (-0.2564) × RAS-associated protein 15 (*RAB15*) (**Figure 3D**). Using the median risk score as the threshold, patients were stratified into high-risk (score ≥ median) and low-risk (score < median) groups. Notably, all coefficients were negative, indicating that higher expression of these five genes correlates with lower risk scores. This recalibration clarifies that elevated expression of these genes confers protective effects, consistent with low-risk group association. Kaplan‒Meier analysis revealed notably poorer OS in the high-risk cohort than in the low-risk cohort within the GSE39582 cohort (median OS: 102.0 months vs. 183.0 months, P < 0.0001; **Figure 3E**). Consistent findings were noted in three independent validation cohorts: the high-risk population displayed worse OS in the GSE17536 and COAD datasets (P = 0.026 and P = 0.0021), with a similar trend noted in the READ set (P = 0.069; **Figures 3F-H**). The risk score distributions, survival rates, and gene expression profiles across the CRC cohort were presented in **Figures 3I-L**. An increasing trend in mortality was noted with increasing risk scores. These findings confirm the robustness and prediction performance of the LLPS-associated gene signature across CRC datasets.

### A nomogram model based on the risk score was established to predict patient prognosis

**Figure 4:**
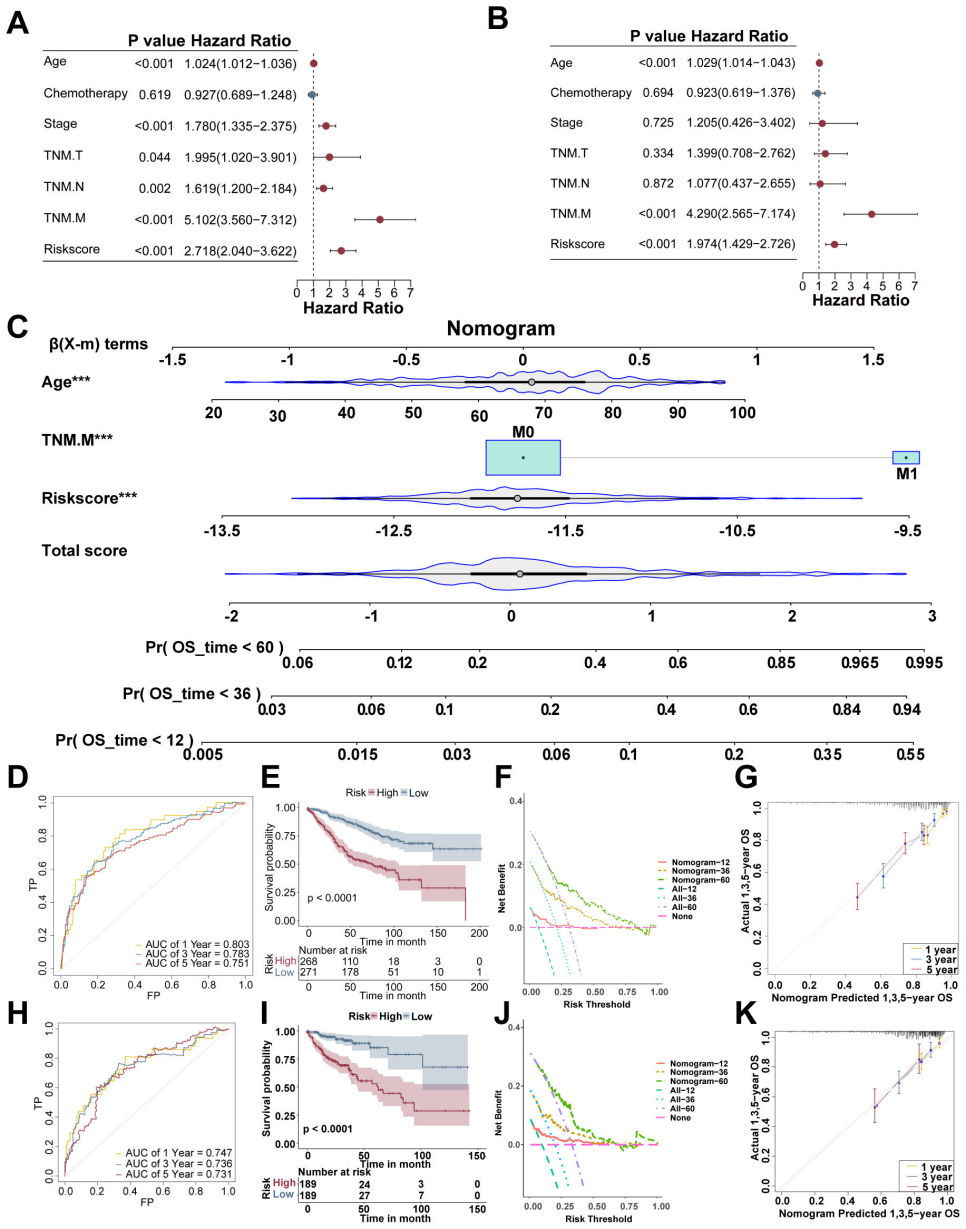
Establishment of the prognostic nomogram model for CRC. Univariate **(A)** and multivariate Cox analyses **(B)** revealed significant prognostic factors in the GSE39582 dataset. **(C)** The nomogram model based on age, M classification, and the risk score was used to predict survival. **(D)** ROC curves displaying different AUCs of the nomogram model. **(E)** Kaplan–Meier survival curves revealed differences in OS between the high- and low-risk cohorts. **(F)** DCA was used to assess the clinical applicability of the nomogram model. **(G)** Calibration curves indicating the agreement between the predicted and actual values. ROC **(H)**, Kaplan–Meier survival **(I)**, DCA **(J)**, and calibration **(K)** curves revealed the ability of the nomogram model to predict the prognosis of the COAD cohort.

In the GSE39582 cohort, the prognostic significance of the risk score and other clinical variables, such as age, chemotherapy status, clinical stage, and TNM classification, was evaluated via univariate and multivariate Cox regression analyses. The data revealed age, M classification, and the LLPS-based risk score as independent prognostic indicators in comparison with other clinical parameters (**Figures 4A, B**). A nomogram model integrating age, M classification, and the risk score was created to forecast the CRC population in the GSE39582 cohort (**Figure 4C**). The AUCs for the nomogram were 0.803, 0.783, and 0.751 for 1-, 3-, and 5-year OS, respectively (**Figure 4D**). The disparity in survival outcomes was marked across the high- and low-risk cohorts (P < 0.0001; **Figure 4E**). DCA indicated greater net clinical benefit in predicting 3- and 5-year OS than in predicting 1-year OS (**Figure 4F**). The calibration curves demonstrated a high level of agreement between the predicted and real survival probabilities (**Figure 4G**). The prediction precision of the nomogram was confirmed in the external COAD cohort, where the model effectively predicted OS (**Figures 4H-K**). Therefore, the LLPS-based nomogram possesses strong predictive power and clinical utility for the prognostic assessment of CRC patients.

### Different patterns of somatic mutations were identified in high- and low-risk cohorts

**Figure 5:**
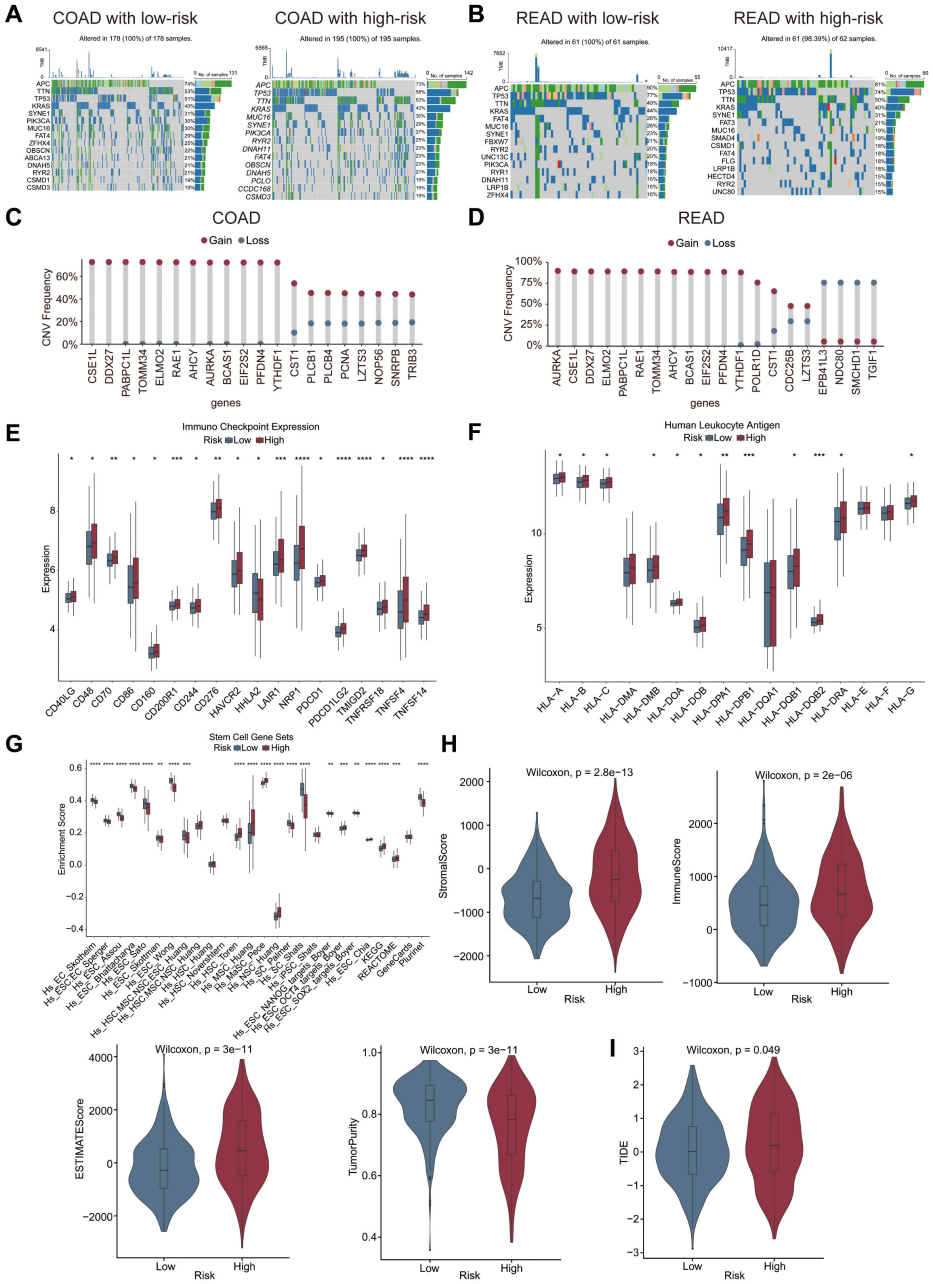
Exploration of the TME. Differences in the somatic mutation patterns of the top 15 genes across the two risk cohorts in the COAD cohort **(A)** and READ cohort **(B)**. CNV frequency of the top 20 genes in the COAD cohort **(C)** and READ cohort **(D)**. Differences in immune checkpoint expression **(E)**, HLA expression **(F)**, stemness enrichment scores **(G)**, immune, stromal, and ESTIMATE scores, and tumor purity **(H)**, and TIDE score **(I)** between the two risk groups in the GSE39582 dataset. * p<0.05; ** p<0.01; *** p<0.001; **** p<0.0001.

The somatic mutations of 430 LLPS-related genes were analyzed across two risk cohorts within the COAD and READ cohorts. Mutation frequencies were comparable across cohorts, with *APC*, *TP53*, *TTN*, and *KRAS* emerging as the most frequently mutated genes (**Figures 5A, B**). In contrast, CNV frequencies demonstrated notable differences between the COAD and READ cohorts. In COAD, copy number amplifications were predominantly noted in *CSE1L*, *DDX27*, and *PABC1L*, whereas deletions were more frequent in *TRIB3*, *SNRPB*, and *NOP56* (**Figure 5C**). In READ, copy number amplifications were more common in *AURKA, CSE1L*, and *DDX27*, whereas deletions were more prevalent in *TGIF1*, *SMCHD1*, and *NDC80* (**Figure 5D**).

### Differences in the TME were observed between the high- and low-risk cohorts

Marked disparities in the TME were noted between the two risk groups. Most immune checkpoint genes, with the exception of HHLA2, were markedly upregulated in the high-risk cohort, indicating possible variations in sensitivity to immune checkpoint inhibitors (ICIs) across risk strata (**Figure 5E**). A similar pattern was noted in the expression of HLA genes, with the majority being upregulated in the high-risk group (**Figure 5F**). Stemness analyses based on 26 gene sets were shownin **Figure 5G**. Analysis via the ESTIMATE algorithm revealed higher immune, stromal, and ESTIMATE scores, along with lower tumor purity, in the high-risk cohort (**Figure 5H**). Furthermore, TIDE score was markedly higher in the high-risk cohort than in the low-risk cohort (**Figure 5I**).

**Figure 6:**
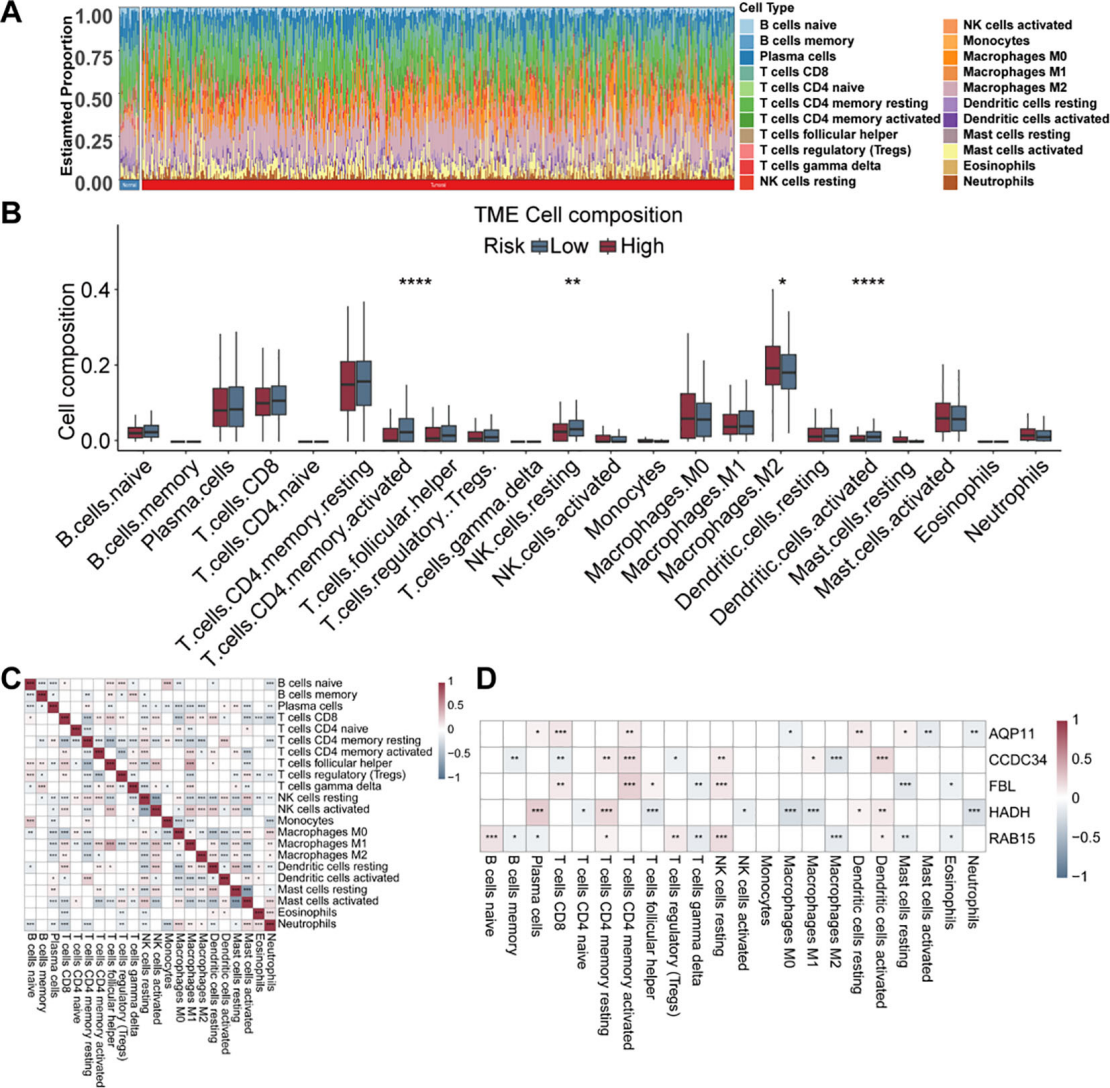
Immune cell infiltration analysis via the CIBERSORT method. **(A)** The stacked histogram displays the immune cell proportions between the CRC and control cohorts. **(B)** Comparison of 22 kinds of immune cells between the high- and low-risk cohorts. **(C)** Heatmap showing the correlations among 22 types of immune cells. **(D)** Heatmap revealing the associations of the five prognostic genes with 22 types of immune cells. * p<0.05; ** p<0.01; *** p<0.001; **** p<0.0001.

Significant differences via the CIBERSORT algorithm were found in the proportions of 22 immune cell types between tumor and control samples in the GSE39582 cohort (**Figure 6A**). Notably, M2 macrophages were substantially enriched in the high-risk cohort, whereas activated memory CD4+ T cells, resting NK cells, and activated dendritic cells were more abundant in the low-risk cohort (**Figure 6B**). Correlation analyses revealed strong relationships among immune cell populations; for example, a marked positive relationship between follicular helper T cells and M1 macrophages was detected (r = 0.449), and a notable negative relationship was detected between activated mast cells and resting mast cells (r = -0.661) (**Figure 6C**). Additionally, the expression of the five prognostic genes was strongly linked to the proportions of various immune cell types (**Figure 6D**). The high expression of inhibitory immune genes and the infiltration of immunosuppressive cells may contribute to a greater likelihood of immune escape, poor immune response, and ultimately unfavorable prognosis in the high-risk group. These findings highlight substantial TME heterogeneity between risk cohorts, which may lead to the development of more individualized and effective antitumour therapeutic strategies.

### Different biological mechanisms drive tumor progression in high- and low-risk groups

**Figure 7:**
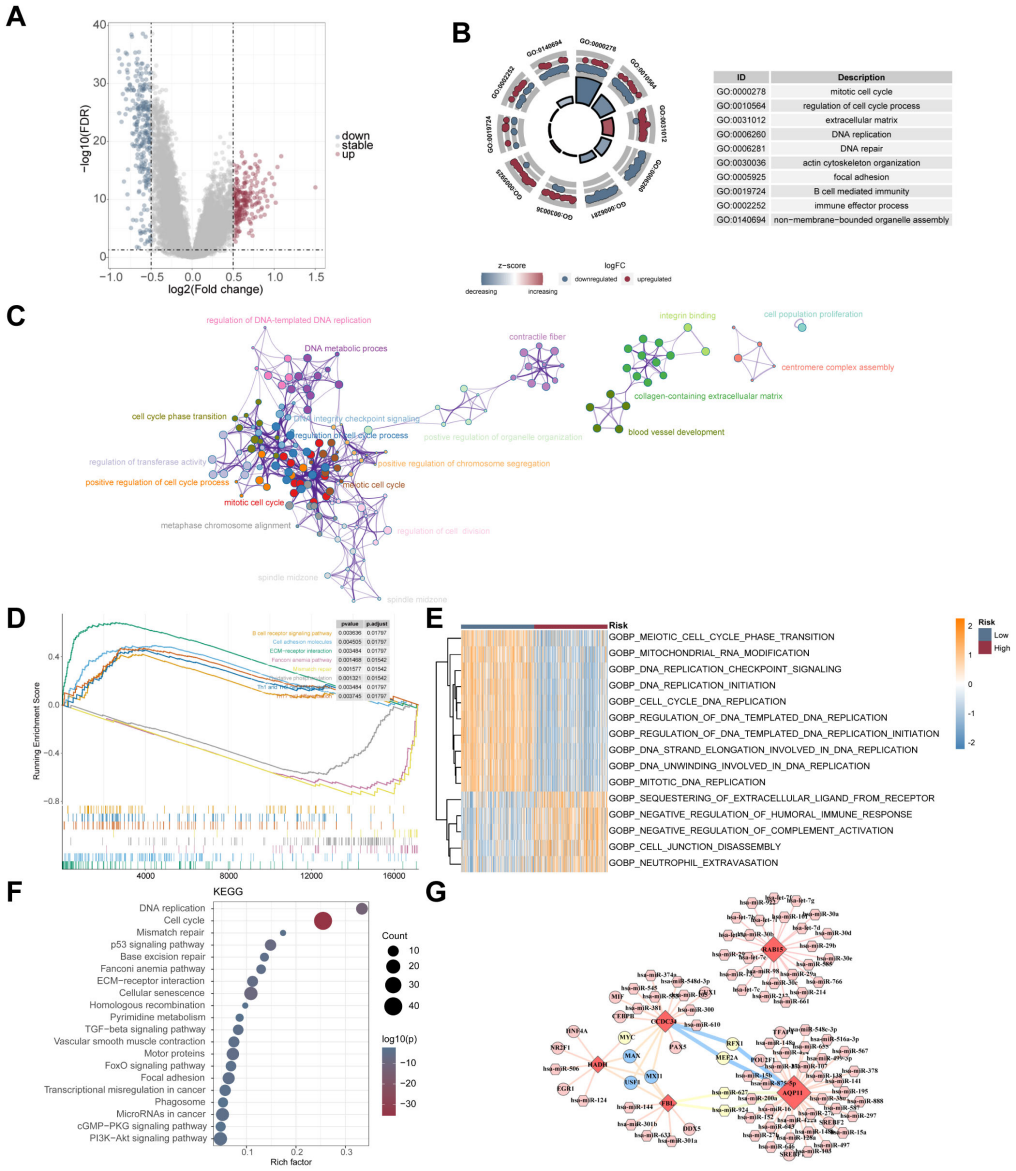
Biological functional enrichment analysis of DEGs across high- and low-risk groups in the GSE39582 dataset. **(A)** The volcano plot represents 492 DEGs, with 230 upregulated genes (red dots) and 262 downregulated genes (blue dots). GO **(B)**, enrichment via Metascape **(C)**, GSEA **(D)**, GSVA **(E)**, and KEGG **(F)** analyses of DEGs. **(G)** Network diagram of the interactions among five prognostic genes, TFs, and miRNAs. The diamonds, hexagons, and circles represent the five prognostic genes, miRNAs, and TFs, respectively.

To elucidate the underlying biological mechanisms distinguishing the high- and low-risk cohorts, DEGs were identified, and 230 upregulated and 262 downregulated genes were identified (**Figure 7A**). GO enrichment analysis revealed their involvement in mitotic cell cycle regulation, cell cycle control, extracellular matrix components, DNA replication, and DNA repair (**Figure 7B**). Functional annotation via the Metascape database further confirmed enrichment in similar processes, such as the mitotic cell cycle, modulation of the cell cycle, collagen-containing extracellular matrix, DNA metabolic process, and positive cell cycle progression regulation (**Figure 7C**). GSEA revealed that pathways such as ECM-receptor interaction, cell adhesion, and B-cell receptor signalling, as well as Th1, Th2 and Th17 cell differentiation, were predominantly enriched in the high-risk cohort. Conversely, the Fanconi anaemia pathway, mismatch repair, and oxidative phosphorylation were enriched in the low-risk population (**Figure 7D**). GSVA revealed further enrichment in the low-risk population of pathways such as meiotic cell cycle phase transition, mitochondrial RNA modification, DNA replication checkpoint signalling, DNA replication initiation, and cell cycle DNA replication. In contrast, pathways such as sequestration of extracellular ligands, negative regulation of the humoral immune response, inhibition of complement activation, cell junction disassembly, and neutrophil extravasation were also notably enriched in the high-risk population of pathways (**Figure 7E**). KEGG pathway analysis revealed the main links of the DEGs to DNA replication, cell cycle regulation, mismatch repair, the p53 signalling pathway, and base excision repair (**Figure 7F**). Comprehensive regulatory network analysis utilizing the NetworkAnalyst database identified several TFs and miRNAs as key regulators of the five prognostic genes. For example, HADH was closely linked to hsa-miR-124 and EGR1; CCDC34 with hsa-miR-381 and CEBPB; FBL with hsa-miR-144 and DDX5; AQP11 with hsa-miR-380 and POU2F1; and RAB15 with hsa-miR-98 (**Figure 7G**).

### ScRNA-seq analysis revealed different distributions and expression patterns of the five prognostic genes

**Figure 8:**
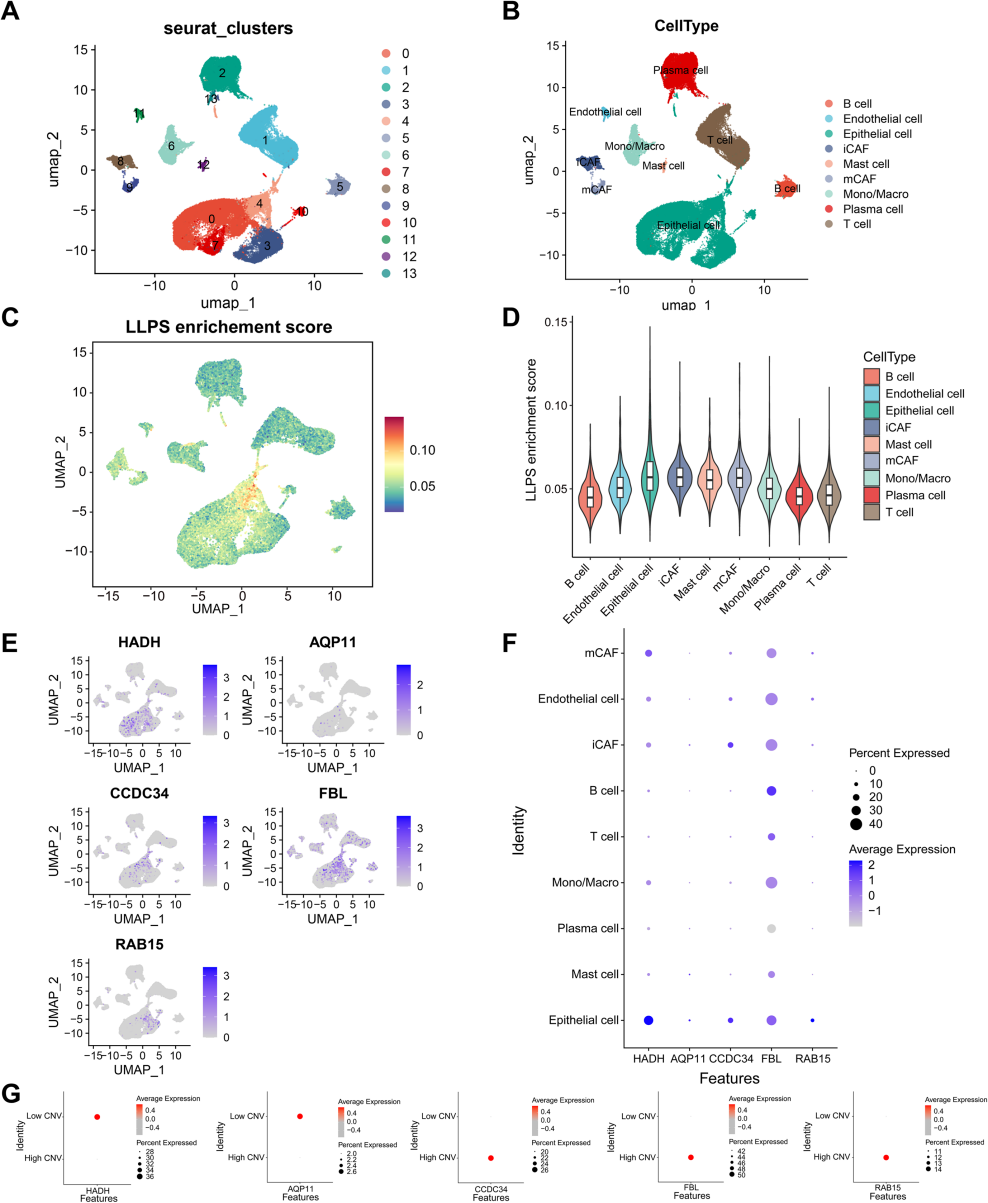
ScRNAseq analysis of the GSE166555 dataset. The UMAP plots revealed thirteen clusters **(A)** and nine types of immune cells **(B)**. **(C)** AUC scores of modules containing 430 LLPS-linked DEGs in each microregion. **(D)** Violin plot displaying LLPS enrichment scores for the nine types of immune cells. **(E)** UMAP plots showing the distribution of the five prognostic genes among the nine types of immune cells. **(F)** Dot plot illustrating the expression of five prognostic genes in the nine types of immune cells. **(G)** The expression of five prognostic genes in the low- and high-CNV groups.

The scRNA-seq data from the GSE166555 dataset were analysed, with 13 distinct cell clusters identified and classified into nine cell types, including B cell, endothelial cell, epithelial cell, inflammatory cancer-associated fibroblast (iCAF)cell, mast cell, myofibroblastic cancer-associated fibroblast cell (mCAF), monocyte/macrophage, plasma cell, and T cell (**Figures 8A, B**). LLPS enrichment scores were markedly higher in epithelial cells than in the other eight cell types (**Figures 8C, D**), indicating that LLPS-related DEGs were predominantly expressed in epithelial cells. The expression and distribution of *AQP11*, *CCDC34*, *FBL*, *HADH*, and *RAB15* were visualized across these immune cell types (**Figures 8E, F**). UMAP and dot plot analyses revealed widespread expression of FBL across all immune cell types except plasma cells. *RAB15* and *HADH* were preferentially expressed in epithelial cells, and *CCDC34* was expressed mainly in inflammatory cancer-associated fibroblasts and epithelial cells. Moreover, *AQP11* and *HADH* were predominantly expressed in the low-CNV group, whereas *CCDC34*, *FBL*, and *RAB15* were enriched in the high-CNV group, suggesting that the former were primarily associated with low-grade malignant cells, and the latter with highly malignant cells (**Figure 8G**).

### Spatial transcriptome analysis revealed different spatial distributions and expression patterns of the five prognostic genes

**Figure 9:**
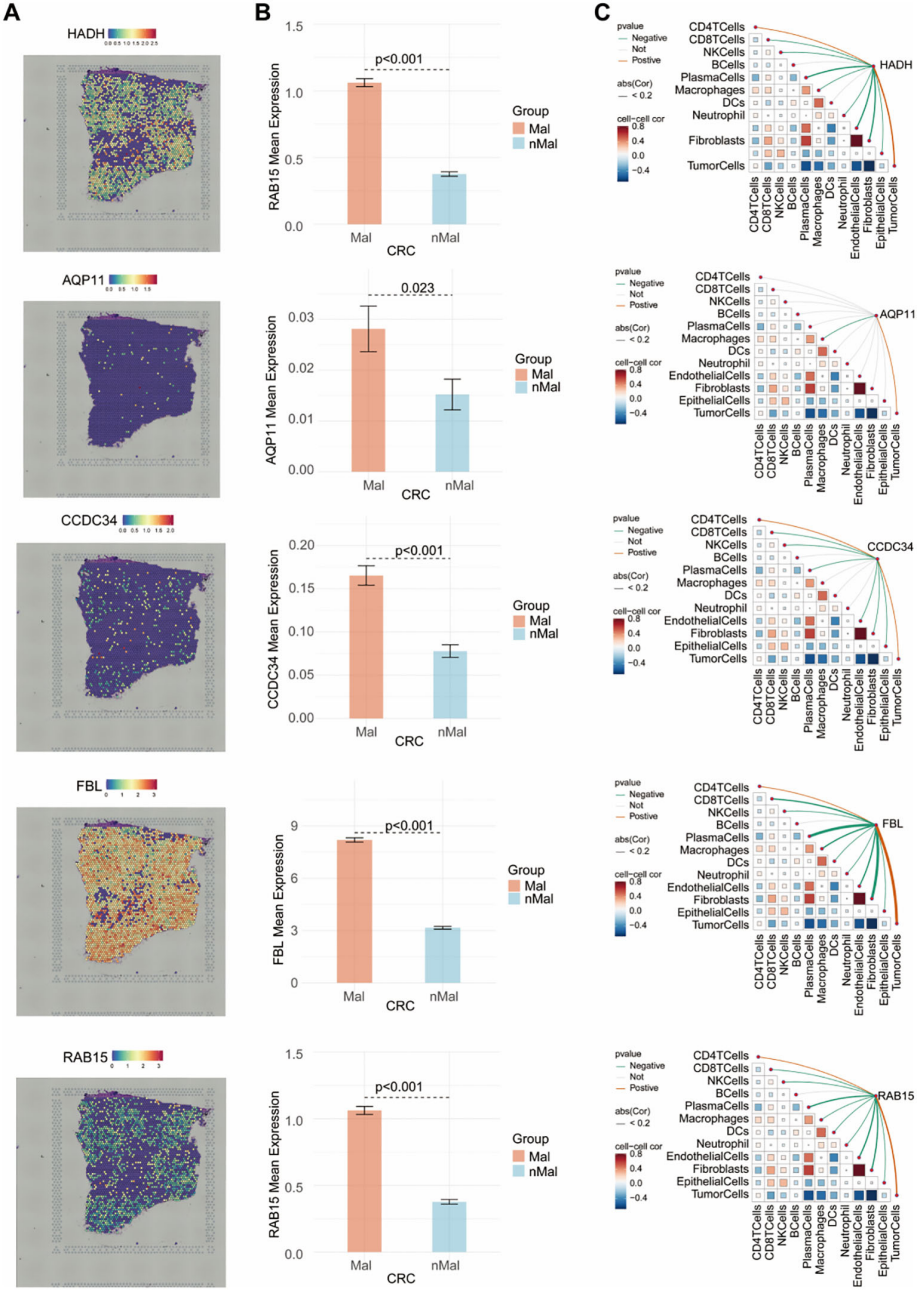
Spatial transcriptomics analysis of five prognostic genes in CRC pathological sections. **(A)** Spatial localization of the five genes in each microregion. **(B)** The expression of the five genes in the malignant and nonmalignant cohorts. **(C)** Links of the five genes to different cell types.

Integrated spatial transcriptome sequencing analysis was conducted via the Sparkle platform to investigate the spatial distribution and expression patterns of five prognostic genes in CRC tissues. Hematoxylin and eosin staining of the pathological sections was illustrated in **Supplementary Figure S1A**. Ten cell types were identified, namely B cell, CD4 T cell, dendritic cell (DC), endothelial cell, epithelial cell, fibroblast, macrophage, neutrophil, plasma cell, and tumor cell (**Supplementary Figure S1B**). The distribution of the five prognostic genes across microregions was shown in **Figure 9A**. The tissue sections were further segmented into malignant and non-malignant regions (**Supplementary Figure S1C**), and AUC scores of 430 LLPS-related genes across all regions were calculated (**Supplementary Figure S1D**). The results revealed higher AUC values for these genes in malignant regions, suggesting their predominant distribution in tumor areas. Correlation analysis further confirmed a significant positive association between the AUC values of these genes and tumor cell content (**Supplementary Figure S1E**). Moreover, the expression levels of the five prognostic genes were markedly higher in malignant regions compared to non-malignant regions (**Figure 9B**). Correlation analysis demonstrated a strong positive relationship between the expression of these five prognostic genes and tumor cell abundance (**Figure 9C**). Similar results were observed in two additional tissue sections, as shown in **Supplementary Figures S2**–**S5**.

### Small molecule compounds screened for CRC treatment

**Figure 10:**
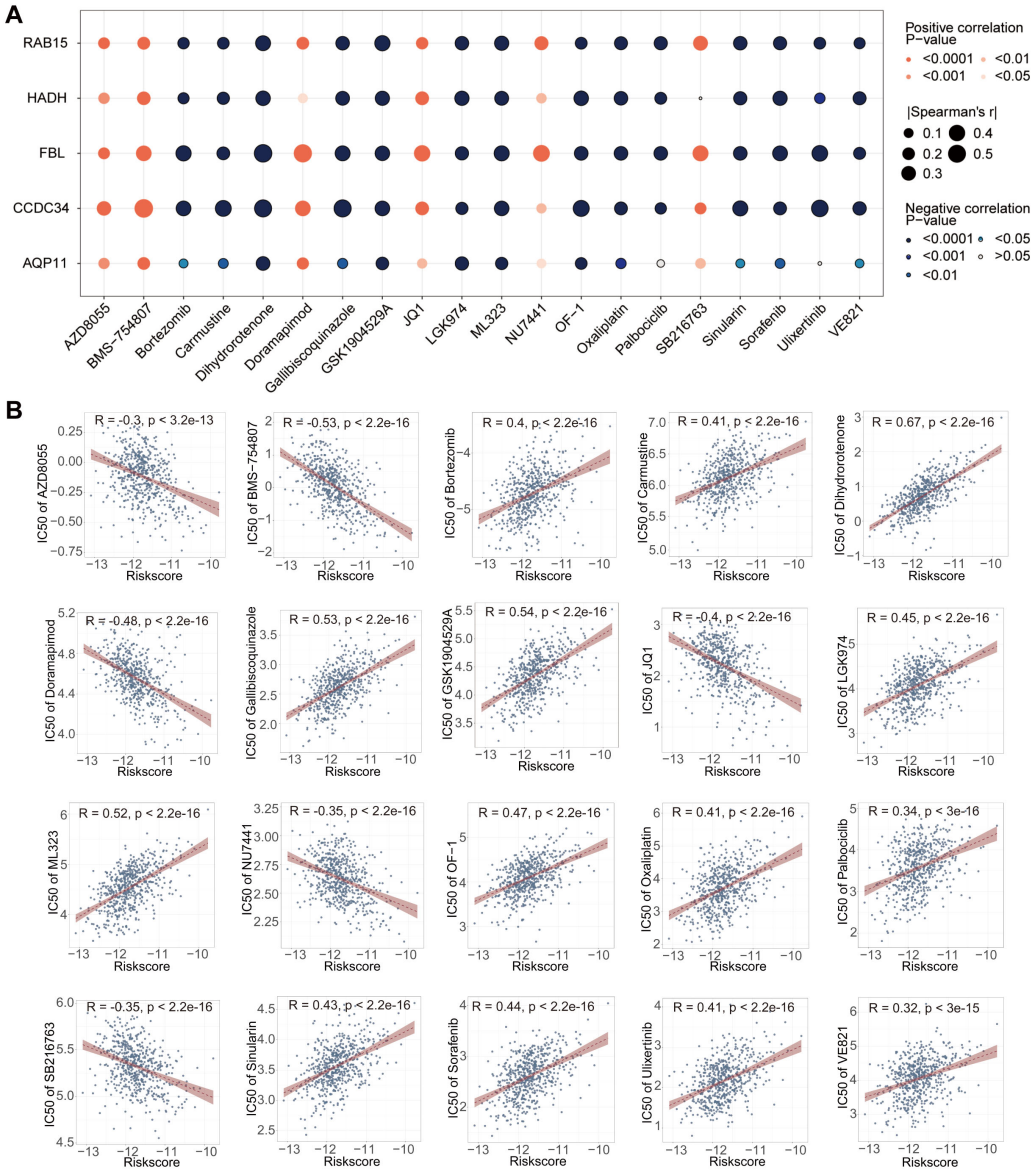
Identification of small-molecule compounds for CRC treatment. **(A)** Heatmap showing the correlations between the expression of the five prognostic genes and the IC50 values of the 20 compounds. The red and blue dots represent positive and negative relationships, respectively. The size of the dots represents the value of Spearman’s r coefficient. **(B)** Scatter plots showing the links between the risk scores and the IC50 values of 20 representative compounds.

Among the 198 candidate small-molecule drugs, 162 presented marked disparities across high- and low-risk individuals. We screened 162 drugs on the basis of the correlation (r > 0.3; p < 0.05) of their IC50 values with the risk score, which yielded 59 candidates. Subsequently, 20 of these drugs, which were deemed potentially effective for CRC treatment, were selected for further analysis. A heatmap revealed strong relationships between the expression of the five prognostic genes and the IC50 values of the selected compounds in the CRC population (**Figure 10A**). The risk scores were negatively related to the IC50 values of AZD8055, BMS-754807, doramapimod, JQ1, NU7441, and SB216763, indicating reduced IC50 values in high-risk patients. Conversely, positive relationships were noted between the risk scores and the IC50 values of 14 other compounds, namely, bortezomib, carmustine, dihydrorotenone, gallibiscoquinazole, GSK1904529A, LGK974, ML323, OF-1, oxaliplatin, palbociclib, sinularin, sorafenib, ulixertinib, and VE821, suggesting reduced IC50 values in low-risk individuals (**Figure 10B**; **Supplementary Figure S6**). The chemical structures of the 20 compounds were obtained from ChemSpider and plotted in **Supplementary Figure S7**.

### *HADH* expression-related enrichment pathways

**Figure 11:**
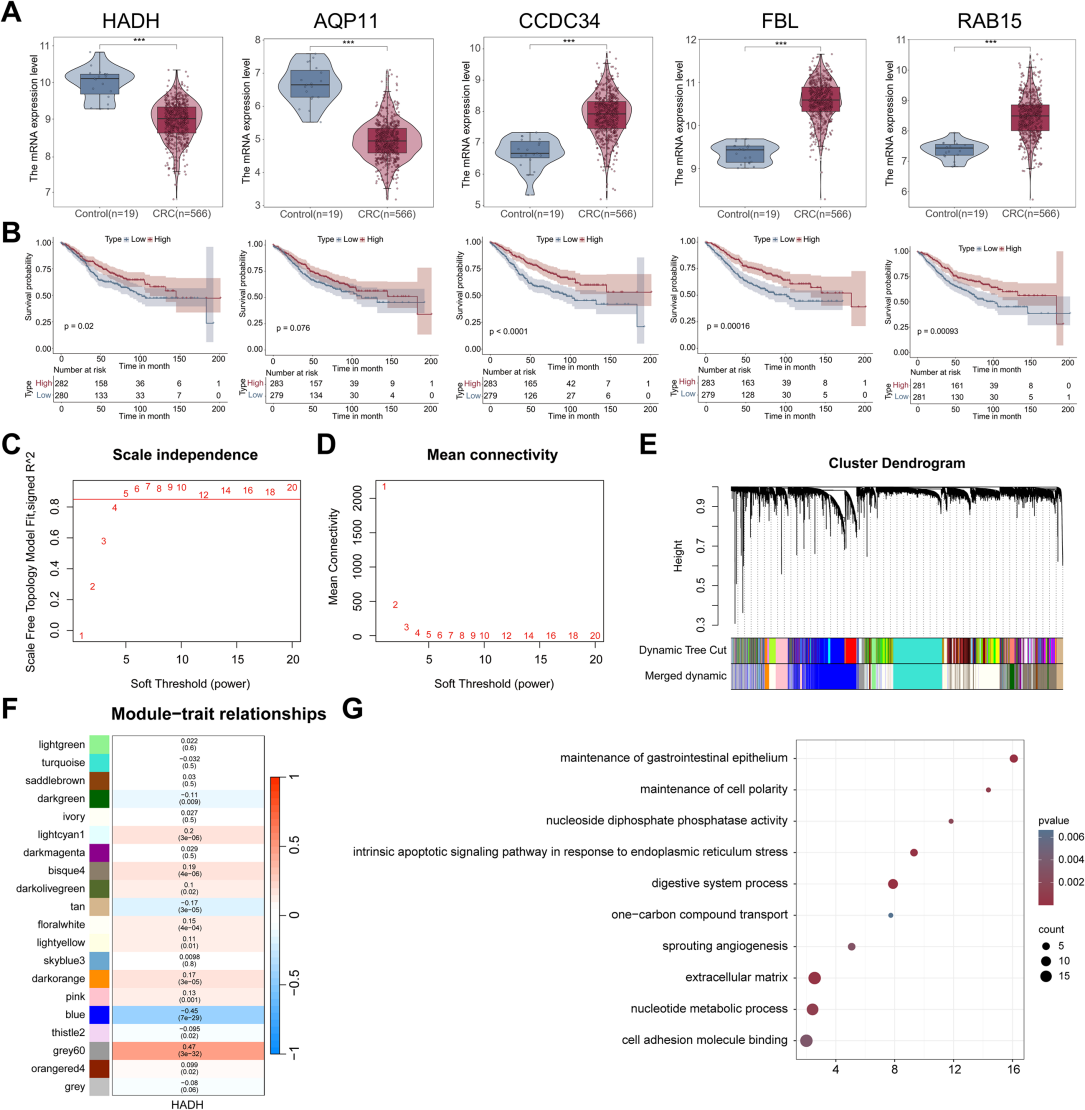
HADH expression-related enrichment pathways via WGCNA. **(A)** The expression of five prognostic genes in the tumor and control cohorts in the GSE39582 dataset. **(B)** Survival comparison between the high- and low-expression cohorts for five genes in the GSE39582 dataset. The optimal soft threshold (β = 5) was determined via the scale-free topology model **(C)** and average connectivity **(D)**. **(E)** Clustering dendrogram demonstrating module feature genes. **(F)** Heatmap revealing the relationships between module feature genes and HADH expression. Correlations (upper) and p values (bottom) were presented. The gray60 module presented the greatest positive correlation with HADH expression and was identified as a key module for HADH expression. **(G)** HADH expression-related enrichment pathways were presented via the Metascape website. *** p<0.001.

Analysis of the GSE39582 dataset revealed notably elevated expression of *CCDC34*, *FBL*, and *RAB15* in tumor tissues, whereas *AQP11* and *HADH* were notably downregulated (**Figure 11A**). High expression levels of *HADH*, *CCDC34*, *FBL*, and *RAB15* were linked to markedly improved OS compared with their respective low-expression cohorts. For *AQP11*, a similar trend toward better survival in the high-expression group was noted (**Figure 11B**).

Our comprehensive findings demonstrated that *HADH* expression was significantly lower in tumor tissues than in normal tissues. Spatial and single-cell analyses further revealed that within the TME, *HADH* was predominantly expressed in tumor epithelial cells with low copy number variation rather than in immune or stromal cells. Patients in the high-*HADH* expression subgroup presented a markedly more favourable prognosis than did those in the low-*HADH* expression subgroup. These results suggest that increasing *HADH* levels in tumor cells may suppress malignant proliferation and improve clinical outcomes in CRC, positioning *HADH* as a potential therapeutic target. On the basis of this integrative evidence, we selected *HADH* for further functional investigation.

The most relevant gene modules related to *HADH* expression were analysed via WGCNA. The optimal soft threshold power was set to 5 (**Figures 11C, D**). Twenty modules were generated via this soft threshold, and a clustering dendrogram of the modules was presented in **Figure 11E**. The correlations between *HADH* expression and gene modules were shown in **Figure 11F**. The results revealed that the gray 60 module presented the greatest significant positive correlation with *HADH* expression (451 genes, r = 047, p = 3e-12). A total of 451 genes in the gray 60 module were imported into the Metascape website for enrichment analysis. The data revealed that *HADH* was enriched mainly in the maintenance of the gastrointestinal epithelium, nucleoside diphosphate phosphatase activity, the intrinsic apoptotic signalling pathway in response to endoplasmic reticulum stress, the extracellular matrix, etc. (**Figure 11G**).

### Experimental verification of *HADH* expression in CRC

**Figure 12:**
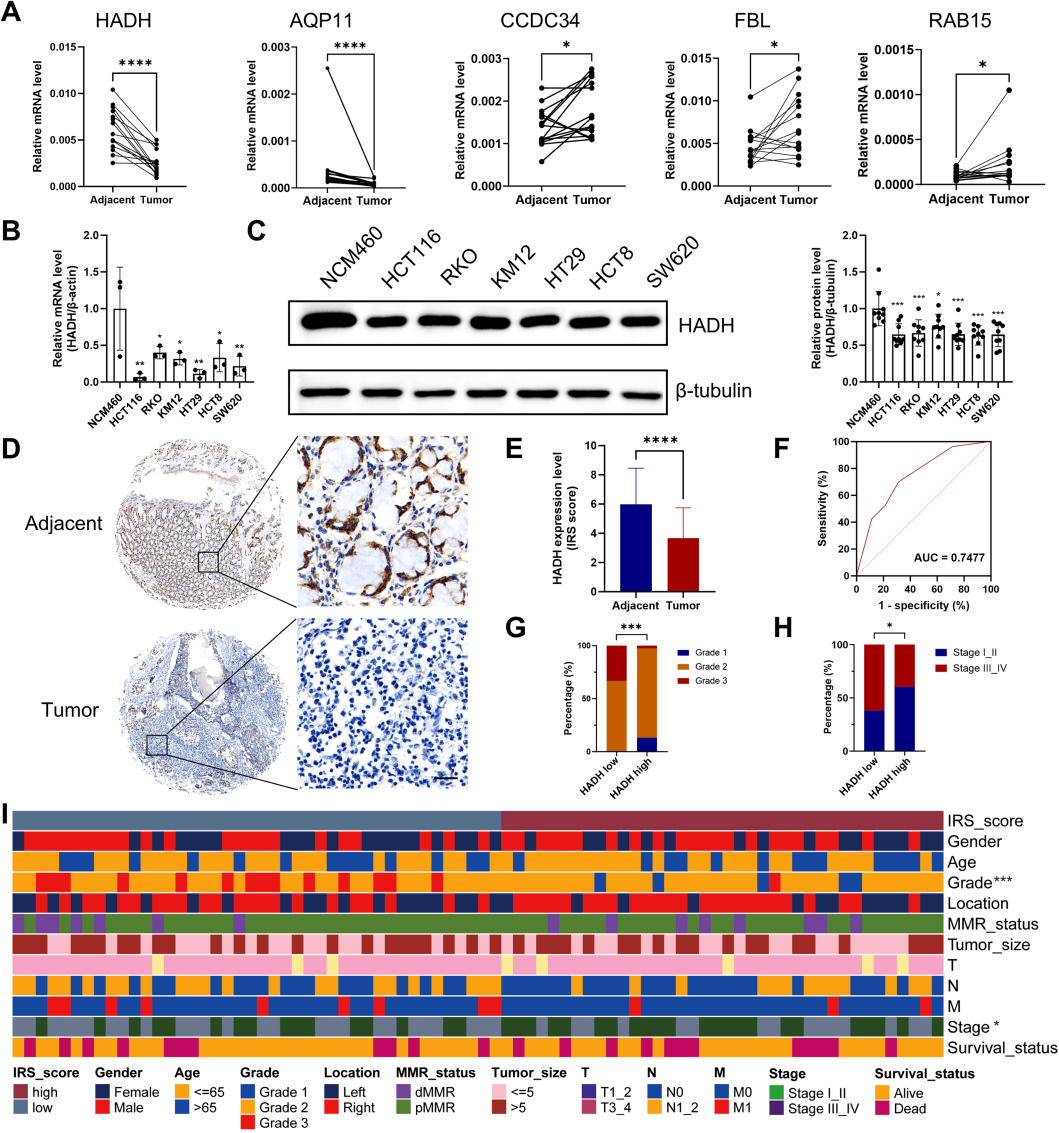
Validation of the expression of prognostic genes for CRC. **(A)** Relative mRNA expression of five genes in fifteen pairs of tumor and paracancerous tissue microarrays. **(B)** Relative HADH mRNA expression in cell lines. **(C)** HADH protein expression in cell lines. **(D)** HADH IHC staining of 80 pairs of tumor and paracancerous tissues from CRC patients. Scale bar, 25 μm. **(E)** IRS scores of IHC staining. **(F)** ROC curve for the CRC diagnosis of the HADH IRS. Comparison of the histological grade **(G)** and clinical stage **(H)** of tumor tissues in the low and high HADH expression subcohorts. **(I)** Heatmap showing the clinical and pathological features of 80 tumor tissues. *p < 0.05; **p < 0.01; ***p < 0.001; ****p < 0.0001.

RT-qPCR analysis of 15 paired tumor and adjacent healthy tissue samples confirmed the differential expression of five prognostic genes (**Figure 12A**). Subsequent validation revealed significantly lower *HADH* expression in HCT116, RKO, KM12, HT29, HCT8, and SW620 cells than in NCM460 cells (**Figures 12B, C**). IHC staining of tumor and adjacent tissues from 80 CRC patients revealed lower HADH protein expression in tumor tissues (**Figures 12D, E**). The diagnostic performance of HADH for CRC, as measured by the AUC, was 0.7477 (**Figure 12F**). High HADH expression was further linked to a lower histological grade and earlier clinical stage (**Figures 12G, H**). The clinical and pathological characteristics of the patients stratified by HADH expression were summarized in **Figure 12I** and **Supplementary Table S4**.

### HADH overexpression inhibited the viability of CRC cells

**Figure 13:**
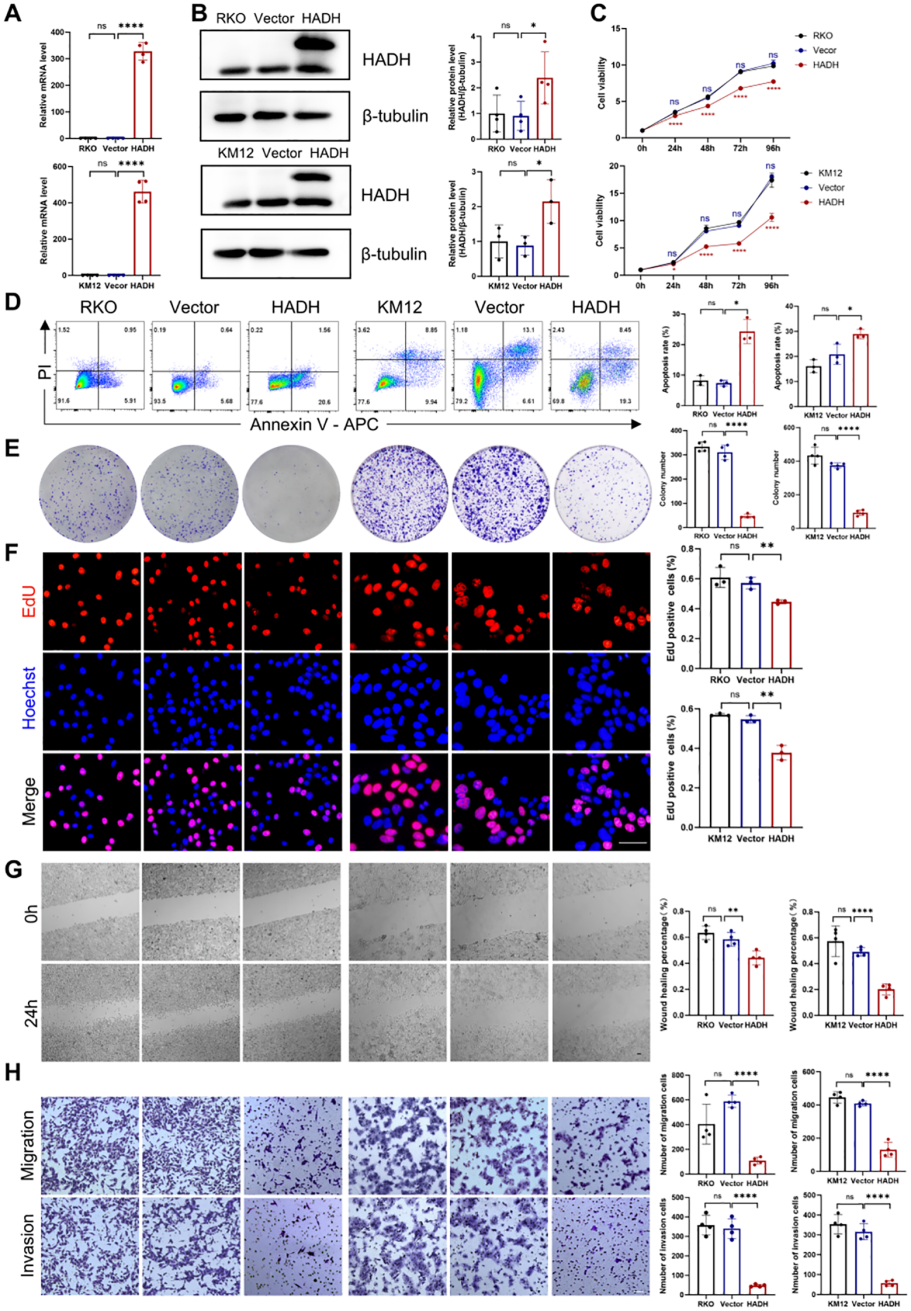
Effects of HADH overexpression on RKO and KM12 cells. **(A)** RT–qPCR results showing increased HADH mRNA levels in the HADH-overexpressing cohorts. **(B)** Western blotting revealed elevated protein levels of HADH in the HADH-overexpressing cohorts. **(C)** CCK8 assay showing decreased cell viability in the overexpression cohorts. **(D)** Flow cytometry results indicating an increased apoptosis rate in the overexpression cohorts. **(E)** A colony formation assay revealed decreased colony formation ability in the overexpression cohorts. **(F)** An EdU assay demonstrated decreased cell proliferation in the overexpression cohorts. Scale bar, 50 μm. **(G)** Wound healing assay showing decreased cell migration in the overexpression cohorts. Scale bar, 200 μm. **(H)** Transwell assays revealed decreased cell migration and invasion in the overexpression cohorts. Scale bar, 200 μm. RKO or KM12: wild-type group; Vector: vector control group; HADH: HADH overexpression group. *p < 0.05; **p < 0.01; ****p < 0.0001. ns, P > 0.05.

HADH function was elucidated via overexpression experiments in the RKO and KM12 cell lines. HADH overexpression was confirmed via RT-qPCR and Western blotting (**Figures 13A, B**). CCK-8 assays revealed decreased cell viability following HADH overexpression (**Figure 13C**). Flow cytometry analysis indicated a marked increase in apoptosis (**Figure 13D**). Colony formation and EdU incorporation assays confirmed the reduced proliferative capacity of HADH-overexpressing cells (**Figures 13E, F**). Furthermore, wound healing and transwell assays revealed marked suppression of cell migration and invasion upon HADH overexpression (**Figures 13G, H**).

## Discussion

Emerging evidence reveals that LLPS regulates biological processes through biomolecular condensates, while its dysregulation contributes to pathologies such as oncogenic signaling activation, genomic instability, and TME remodeling (36). The identification of LLPS-linked biomarkers may provide insights for tumor subtyping and prognostic assessment. Our study investigated how LLPS influences CRC. On the basis of the expression profiles of the 430 LLPS-associated DEGs, five prognostic genes were identified via univariate Cox regression, LASSO regression and stepAIC algorithms, culminating in the construction of a nomogram model. The derived risk scores exhibited strong associations with drug sensitivity, clinical outcomes, tumor stemness, the TME, clinicopathological features, and genomic alterations.

Our results revealed that the 430 LLPS-related DEGs were enriched primarily in pathways related to the cell cycle, DNA replication, and DNA damage repair. Dysregulated LLPS can repair DNA damage in tumor cells induced by radiotherapy and chemotherapy, leading to treatment failure. For example, under physiological conditions, SUMOylated RNF168 is inefficiently recruited to DSB sites, thereby impairing H2A ubiquitination and 53BP1 accumulation within nuclear condensates, ultimately reducing DNA repair efficiency. In CRC, the overexpression of SENP1 decreases RNF168 SUMOylation, enhances DNA repair capacity, and correlates with poor prognosis (37). Notably, the LLPS-based prognostic model revealed a markedly worse overall prognosis in high-risk individuals than in low-risk individuals. Compounds such as JQ-1 and BMS-754807 may serve as promising therapeutic candidates for high-risk individuals, whereas agents such as gefitinib, sorafenib, and oxaliplatin may exhibit greater efficacy in low-risk individuals. These findings prove the importance of LLPS-related genes in CRC progression and present promising avenues for personalized treatments.

Tumor immune evasion and resistance to ICIs remain major obstacles in cancer immunotherapy. Recent studies suggest that LLPS modulates immune cell infiltration within the TME. For example, LLPS-mediated disruption of T-cell receptor signaling by upregulated LAG3 inhibits T-cell proliferation and differentiation, thereby promoting an immunosuppressive milieu (38). IFN-γ-triggered phase separation of the KAT8-IRF1 complex enhances PD-L1 expression and facilitates immune evasion (39). In the present analysis, notable disparities in immune checkpoint expression, HLA levels, immune cell infiltration, and genomic mutation profiles were noted between risk cohorts. Additionally, higher immune and stromal scores, alongside lower tumor purity, were noted in the high-risk group, indicating the presence of more extensive immune cells in the TME. Notably, the high-risk group exhibited elevated TIDE scores, suggesting potentially poorer responses to immunotherapy, which may partially account for their unfavorable prognosis. Overall, LLPS can regulate immune dynamics and is a possible predictive biomarker for the immunotherapeutic response.

Our prognostic model, comprising five genes (*HADH*, *AQP11*, *CCDC34*, *FBL*, and *RAB15*), demonstrated robust predictive ability for patient prognosis, the immune response, and mutational landscapes. AQP11 *AQP11* encodes a water-channel membrane protein that is downregulated in CRC and facilitates tumor cell proliferation, migration, invasion, and adhesion (40). *CCDC34*, a coiled-coil domain-containing protein, is upregulated in CRC and linked to reduced apoptosis and increased metastasis, possibly via the modulation of Bcl-2, survivin, E-cadherin, N-cadherin, and MMP-9 (41). FBL is a highly conserved nucleolar methyltransferase that promotes nucleolar assembly and pre-rRNA synthesis via phase separation, contributing to ribosome biogenesis and cellular proliferation (42). Long non-coding RNA (lncRNA) binds to multiple FBL molecules and partially blocks the glycine- and arginine-rich (GAR) domain, thereby inhibiting excessive solid phase condensation caused by GAR-GAR interactions. This mechanism maintains the liquid phase fluidity of FBL and consequently sustains cancer cell growth (43). *RAB15* is a small GTPase belonging to the RAS superfamily that is predominantly localized to membrane-bound organelles where it participates in vesicle-mediated intracellular transport. It is critical for modulating cell division, migration, and signal transduction in tumor cells, and modulates immune receptor trafficking as well as the secretion of chemokines and cytokines within the immune system (44).

Metabolic reprogramming is a recognized hallmark of cancer progression. *HADH*, a member of the 3-hydroxyacyl-CoA dehydrogenase family, encodes a mitochondrial enzyme essential for fatty acid β-oxidation (45). *HADH* expression is reduced in gastric cancer, where it inhibits PTEN expression and promotes AKT phosphorylation, thereby activating the Akt signaling pathway and facilitating gastric cancer cell migration and invasion (46). Similar tumor-suppressive roles have been reported in renal, esophageal, hepatic, and ampullary adenocarcinomas. In CRC, reduced *HADH* expression is associated with advanced disease stages and poor prognosis (47–49). These effects possibly arise from *HADH* deficiency-induced disruption of fatty acid metabolism and subsequent lipid accumulation, leading to metabolic reprogramming and uncontrolled tumor cell proliferation (50). Our study, on the other hand, supported the idea that *HADH* was significantly reduced in CRC tissues and was more highly expressed in early tumor tissues. Spatial transcriptomic analysis confirmed the predominant expression of *HADH* in tumor cells. Therefore, *HADH* possibly influences CRC progression, although the underlying mechanisms remain to be fully elucidated. *HADH* overexpression experiments revealed that *HADH* inhibited cell proliferation, migration, and invasion while facilitating apoptosis. Hypermethylation of the *HADH* gene has been identified as one mechanism underlying its downregulation in CRC, where it modulates cancer cell proliferation through the Wnt/β-catenin and Wnt/ROR2/DVL2 signaling pathways (46, 51). *HADH* overexpression may suppress tumor cell proliferation by inhibiting NRF2-mediated glutathione synthesis and inducing oxidative damage in cancer cells (52). Therefore, *HADH* can serve not only as a diagnostic biomarker for CRC but is also closely associated with its prognosis. Future studies involving a series of experiments *in vitro* and *in vivo* are warranted to further explore the mechanisms by which HADH regulates CRC progression.

According to infomation from the DrLLPS database, AQP11, CCDC34, HADH, RAB15, and FBL are all classified as client proteins, with AQP11 involved in nucleolus formation, CCDC34 in centrosome/spindle pole body formation, HADH in both nucleolus and postsynaptic density formation, RAB15 in postsynaptic density formation, and FBL in the formation of Cajal bodies, nucleoli, P-bodies, and stress granules. LLPS has previously been shown to explain tumor heterogeneity in gliomas and melanomas. Previous studies have developed numerous prognostic models for CRC based on a variety of molecular signatures, including immune infiltration, tumor metabolism, stemness, non-coding RNAs, epigenetic regulators, and cell death pathways. While each model offers unique insights into tumor biology and demonstrates robust predictive capacity, our LLPS-related 5-gene model emerges as a novel predictor. It demonstrates prognostic accuracy that is comparable, if not superior, to these established signatures. Uniquely, our model links prognosis to the functional integrity of organelles involved in phase separation. By capturing critical features of tumor aggressiveness and undergoing extensive validation, the LLPS-related model exhibits significant clinical utility. Furthermore, its correlation with multiple biological processes—such as ribosome biogenesis, metabolism (specifically *HADH*), and immune modulation—may underpin its superior performance. Moving forward, the integration of molecular signatures with clinicopathological factors appears to be crucial for achieving effective risk stratification. This provides a novel, mechanism-driven perspective for understanding the heterogeneity and malignant progression of CRC, offering new insights for individualized precision therapy in CRC.

Nonetheless, there are several limitations. The prognostic model was developed primarily utilizing open datasets, representing a retrospective analysis based on existing data. The mechanistic contribution of *HADH* to CRC pathogenesis remains incompletely defined. Furthermore, while functional validation was performed for HADH, the other four prognostic genes were not subjected to further experimental exploration. Future studies involving prospective clinical cohorts and mechanistic investigations at the molecular level for all five signature genes are essential to further validate and expand upon these findings.

## Conclusion

In conclusion, an LLPS-based prognostic model was established to stratify CRC patients into two molecular subtypes with distinct clinical outcomes: genomic alterations, immune profiles, and therapeutic sensitivities. This model facilitates a deeper understanding of CRC heterogeneity and may serve as a valuable tool for guiding individualized therapeutic strategies.

## Data Availability

The original contributions presented in the study are included in the article/**Supplementary Material**. Further inquiries can be directed to the corresponding authors.

## Supplementary material

Supplementary Figure 1 Spatial transcriptomics analysis of the CRC pathological section. **(A)** The hematoxylin-eosin staining of the pathologic section. **(B)** The spatial localization of all cell types. Each dot represented a microregion, and different colors represented different cell types. **(C)** Distribution of malignant and nonmalignant regions. **(D)** AUC scores of modules containing 430 LLPS-related genes. **(E)** Correlation between AUC scores and different cell types.

Supplementary Figure 2 Spatial transcriptomics analysis of the second CRC pathological section. **(A)** The spatial localization of the five genes in each microregion. **(B)** The expression levels of the five genes in the malignant and nonmalignant groups. **(C)** The correlation between the five genes and different cell types.

Supplementary Figure 3 Spatial transcriptomics analysis of the second CRC pathological section. **(A)** The hematoxylin-eosin staining of the pathologic section. **(B)** The spatial localization of all cell types. Each dot represented a microregion, and different colors represented different cell types. **(C)** Distribution of malignant and nonmalignant regions. **(D)** AUC scores of modules containing 430 LLPS-related genes. **(E)** Correlation between AUC scores and different cell types.

Supplementary Figure 4 Spatial transcriptomics analysis of the third CRC pathological section. **(A)** The spatial localization of the five genes in each microregion. **(B)** The expression levels of the five genes in the malignant and nonmalignant groups. **(C)** The correlation between the five genes and different cell types.

Supplementary Figure 5 Spatial transcriptomics analysis of the third CRC pathological section. **(A)** The hematoxylin-eosin staining of the pathologic section. **(B)** The spatial localization of all cell types. Each dot represented a microregion, and different colors represented different cell types. **(C)** Distribution of malignant and nonmalignant regions. **(D)** AUC scores of modules containing 430 LLPS-related genes. **(E)** Correlation between AUC scores and different cell types.

Supplementary Figure 6 Comparisons of IC50 of twenty drugs in the high- and low-risk groups. *** p<0.001.

Supplementary Figure 7 Chemical structures of twenty drugs.

Supplementary Table 1 Details of the bulk RNA sequencing datasets from the study.

Supplementary Table 2 The data statistics of 3585 known LLPS-associated proteins.

Supplementary Table 3 Sequences of the primers used for PCR.

Supplementary Table 4 Characteristics between high and low HADH expression subgroups in CRC patients.

## References

[1] Shin Y Brangwynne CP . Liquid phase condensation in cell physiology and disease. Science. (2017) 357:eaaf4382. doi: 10.1126/science.aaf4382, PMID: 28935776

[2] Wang L Zhou W . Phase separation as a new form of regulation in innate immunity. Mol Cell. (2024) 84:2410–22. doi: 10.1016/j.molcel.2024.06.004, PMID: 38936362

[3] Yang J Griffin A Qiang Z Ren J . Organelle-targeted therapies: a comprehensive review on system design for enabling precision oncology. Signal Transduct Target Ther. (2022) 7:379. doi: 10.1038/s41392-022-01243-0, PMID: 36402753 PMC9675787

[4] Bu FT Wang HY Xu C Song KL Dai Z Wang LT . The role of m6A-associated membraneless organelles in the RNA metabolism processes and human diseases. Theranostics. (2024) 14:4683–700. doi: 10.7150/thno.99019, PMID: 39239525 PMC11373618

[5] Banani SF Rice AM Peeples WB Lin Y Jain S Parker R . Compositional Control of Phase-Separated Cellular Bodies. Cell. (2016) 166:651–63. doi: 10.1016/j.cell.2016.06.010, PMID: 27374333 PMC4967043

[6] Zhang Y Seemann J . RNA scaffolds the Golgi ribbon by forming condensates with GM130. Nat Cell Biol. (2024) 26:1139–53. doi: 10.1038/s41556-024-01447-2, PMID: 38992139

[7] Banani SF Lee HO Hyman AA Rosen MK . Biomolecular condensates: organizers of cellular biochemistry. Nat Rev Mol Cell Biol. (2017) 18:285–98. doi: 10.1038/nrm.2017.7, PMID: 28225081 PMC7434221

[8] Tong X Tang R Xu J Wang W Zhao Y Yu X . Liquid-liquid phase separation in tumor biology. Signal Transduct Target Ther. (2022) 7:221. doi: 10.1038/s41392-022-01076-x, PMID: 35803926 PMC9270353

[9] Zhang X Yuan L Zhang W Zhang Y Wu Q Li C . Liquid-liquid phase separation in diseases. MedComm (2020). (2024) 5:e640. doi: 10.1002/mco2.v5.7 39006762 PMC11245632

[10] Nott TJ Petsalaki E Farber P Jervis D Fussner E Plochowietz A . Phase transition of a disordered nuage protein generates environmentally responsive membraneless organelles. Mol Cell. (2015) 57:936–47. doi: 10.1016/j.molcel.2015.01.013, PMID: 25747659 PMC4352761

[11] Wang YL Zhao WW Bai SM Feng LL Bie SY Gong L . MRNIP condensates promote DNA double-strand break sensing and end resection. Nat Commun. (2022) 13:2638. doi: 10.1038/s41467-022-30303-w, PMID: 35551189 PMC9098523

[12] Qin C Wang YL Zhou JY Shi J Zhao WW Zhu YX . RAP80 phase separation at DNA double-strand break promotes BRCA1 recruitment. Nucleic Acids Res. (2023) 51:9733–47. doi: 10.1093/nar/gkad686, PMID: 37638744 PMC10570032

[13] Choi J Rafiq NM Park D . Liquid-liquid phase separation in presynaptic nerve terminals. Trends Biochem Sci. (2024) 49:888–900. doi: 10.1016/j.tibs.2024.07.005, PMID: 39198083

[14] Kiang KM Ahad L Zhong X Lu QR . Biomolecular condensates: hubs of Hippo-YAP/TAZ signaling in cancer. Trends Cell Biol. (2024) 34:566–77. doi: 10.1016/j.tcb.2024.04.009, PMID: 38806345

[15] Lu B Qiu R Wei J Wang L Zhang Q Li M . Phase separation of phospho-HDAC6 drives aberrant chromatin architecture in triple-negative breast cancer. Nat Cancer. (2024) 5:1622–40. doi: 10.1038/s43018-024-00816-y, PMID: 39198689

[16] Zhang J Ni Z Zhang Y Guo Y Zhai R Wang M . DAZAP1 Phase Separation Regulates Mitochondrial Metabolism to Facilitate Invasion and Metastasis of Oral Squamous Cell Carcinoma. Cancer Res. (2024) 84:3818–33. doi: 10.1158/0008-5472.CAN-24-0067, PMID: 39120588

[17] Kim YR Joo J Lee HJ Kim C Park JC Yu YS . Prion-like domain mediated phase separation of ARID1A promotes oncogenic potential of Ewing’s sarcoma. Nat Commun. (2024) 15:6569. doi: 10.1038/s41467-024-51050-0, PMID: 39095374 PMC11297139

[18] Bray F Laversanne M Sung H Ferlay J Siegel RL Soerjomataram I . Global cancer statistics 2022: GLOBOCAN estimates of incidence and mortality worldwide for 36 cancers in 185 countries. CA Cancer J Clin. (2024) 74:229–63. doi: 10.3322/caac.21834, PMID: 38572751

[19] Bian S Hou Y Zhou X Li X Yong J Wang Y . Single-cell multiomics sequencing and analyses of human colorectal cancer. Science. (2018) 362:1060–3. doi: 10.1126/science.aao3791, PMID: 30498128

[20] Dekker E Tanis PJ Vleugels JLA Kasi PM Wallace MB . Colorectal cancer. Lancet. (2019) 394:1467–80. doi: 10.1016/S0140-6736(19)32319-0, PMID: 31631858

[21] Chen RX Xu SD Deng MH Hao SH Chen JW Ma XD . Mex-3 RNA binding family member A (MEX3A)/circMPP6 complex promotes colorectal cancer progression by inhibiting autophagy. Signal Transduct Target Ther. (2024) 9:80. doi: 10.1038/s41392-024-01787-3, PMID: 38565536 PMC10987644

[22] Schmidt HB Jaafar ZA Wulff BE Rodencal JJ Hong K Aziz-Zanjani MO . Oxaliplatin disrupts nucleolar function through biophysical disintegration. Cell Rep. (2022) 41:111629. doi: 10.1016/j.celrep.2022.111629, PMID: 36351392 PMC9749789

[23] Goldman MJ Craft B Hastie M Repečka K McDade F Kamath A . Visualizing and interpreting cancer genomics data via the Xena platform. Nat Biotechnol. (2020) 38:675–8. doi: 10.1038/s41587-020-0546-8, PMID: 32444850 PMC7386072

[24] Marisa L de Reyniès A Duval A Selves J Gaub MP Vescovo L . Gene expression classification of colon cancer into molecular subtypes: characterization, validation, and prognostic value. PloS Med. (2013) 10:e1001453. doi: 10.1371/journal.pmed.1001453, PMID: 23700391 PMC3660251

[25] Smith JJ Deane NG Wu F Merchant NB Zhang B Jiang A . Experimentally derived metastasis gene expression profile predicts recurrence and death in patients with colon cancer. Gastroenterology. (2010) 138:958–68. doi: 10.1053/j.gastro.2009.11.005, PMID: 19914252 PMC3388775

[26] Ning W Guo Y Lin S Mei B Wu Y Jiang P . DrLLPS: a data resource of liquid-liquid phase separation in eukaryotes. Nucleic Acids Res. (2020) 48:D288–d95. doi: 10.1093/nar/gkz1027, PMID: 31691822 PMC7145660

[27] Zheng J Wu Z Qiu Y Wang X Jiang X . An integrative multi-omics analysis based on liquid-liquid phase separation delineates distinct subtypes of lower-grade glioma and identifies a prognostic signature. J Transl Med. (2022) 20:55. doi: 10.1186/s12967-022-03266-1, PMID: 35093128 PMC8800244

[28] Yoshihara K Shahmoradgoli M Martínez E Vegesna R Kim H Torres-Garcia W . Inferring tumour purity and stromal and immune cell admixture from expression data. Nat Commun. (2013) 4:2612. doi: 10.1038/ncomms3612, PMID: 24113773 PMC3826632

[29] Newman AM Liu CL Green MR Gentles AJ Feng W Xu Y . Robust enumeration of cell subsets from tissue expression profiles. Nat Methods. (2015) 12:453–7. doi: 10.1038/nmeth.3337, PMID: 25822800 PMC4739640

[30] Pinto JP Kalathur RK Oliveira DV Barata T MaChado RS MaChado S . StemChecker: a web-based tool to discover and explore stemness signatures in gene sets. Nucleic Acids Res. (2015) 43:W72–7. doi: 10.1093/nar/gkv529, PMID: 26007653 PMC4489266

[31] Feng C Jin X Han Y Guo R Zou J Li Y . Expression and Prognostic Analyses of ITGA3, ITGA5, and ITGA6 in Head and Neck Squamous Cell Carcinoma. Med Sci Monit. (2020) 26:e926800. doi: 10.12659/MSM.926800, PMID: 33099569 PMC7594586

[32] Feng C Zhu L Mao W Dong P Chen X . Expression and prognosis of cellular senescence genes in head and neck squamous cell carcinoma. Holistic Integr Oncol. (2025) 4:2. doi: 10.1007/s44178-024-00115-7

[33] Xia J Benner MJ Hancock RE . NetworkAnalyst–integrative approaches for protein-protein interaction network analysis and visual exploration. Nucleic Acids Res. (2014) 42:W167–74. doi: 10.1093/nar/gku443, PMID: 24861621 PMC4086107

[34] Mayakonda A Lin DC Assenov Y Plass C Koeffler HP . Maftools: efficient and comprehensive analysis of somatic variants in cancer. Genome Res. (2018) 28:1747–56. doi: 10.1101/gr.239244.118, PMID: 30341162 PMC6211645

[35] Maeser D Gruener RF Huang RS . OncoPredict: an R package for predicting *in vivo* or cancer patient drug response and biomarkers from cell line screening data. Brief Bioinform. (2021) 22:bbab260. doi: 10.1093/bib/bbab260, PMID: 34260682 PMC8574972

[36] Zheng LW Liu CC Yu KD . Phase separations in oncogenesis, tumor progressions and metastasis: a glance from hallmarks of cancer. J Hematol Oncol. (2023) 16:123. doi: 10.1186/s13045-023-01522-5, PMID: 38110976 PMC10726551

[37] Wei M Huang X Liao L Tian Y Zheng X . SENP1 Decreases RNF168 Phase Separation to Promote DNA Damage Repair and Drug Resistance in Colon Cancer. Cancer Res. (2023) 83:2908–23. doi: 10.1158/0008-5472.CAN-22-4017, PMID: 37350666

[38] Guy C Mitrea DM Chou PC Temirov J Vignali KM Liu X . LAG3 associates with TCR-CD3 complexes and suppresses signaling by driving co-receptor-Lck dissociation. Nat Immunol. (2022) 23:757–67. doi: 10.1038/s41590-022-01176-4, PMID: 35437325 PMC9106921

[39] Wu Y Zhou L Zou Y Zhang Y Zhang M Xu L . Disrupting the phase separation of KAT8-IRF1 diminishes PD-L1 expression and promotes antitumor immunity. Nat Cancer. (2023) 4:382–400. doi: 10.1038/s43018-023-00522-1, PMID: 36894639 PMC10042735

[40] Zhu X Jin X Li Z Chen X Zhao J . miR-152-3p facilitates cell adhesion and hepatic metastases in colorectal cancer via targeting AQP11. Pathol Res Pract. (2023) 244:154389. doi: 10.1016/j.prp.2023.154389, PMID: 36889174

[41] Qi W Shao F Huang Q . Expression of Coiled-Coil Domain Containing 34 (CCDC34) and its Prognostic Significance in Pancreatic Adenocarcinoma. Med Sci Monit. (2017) 23:6012–8. doi: 10.12659/MSM.907951, PMID: 29257799 PMC5745713

[42] Feric M Vaidya N Harmon TS Mitrea DM Zhu L Richardson TM . Coexisting Liquid Phases Underlie Nucleolar Subcompartments. Cell. (2016) 165:1686–97. doi: 10.1016/j.cell.2016.04.047, PMID: 27212236 PMC5127388

[43] Sun YM Zhu SX Chen XT Pan Q An Y Chen TQ . lncRNAs maintain the functional phase state of nucleolar prion-like protein to facilitate rRNA processing. Mol Cell. (2024) 84:4878–95.e10. doi: 10.1016/j.molcel.2024.10.036, PMID: 39579766

[44] Jiang X Yang L Gao Q Liu Y Feng X Ye S . The Role of RAB GTPases and Its Potential in Predicting Immunotherapy Response and Prognosis in Colorectal Cancer. Front Genet. (2022) 13:828373. doi: 10.3389/fgene.2022.828373, PMID: 35154286 PMC8833848

[45] Liu Y Lu LL Wen D Liu DL Dong LL Gao DM . MiR-612 regulates invadopodia of hepatocellular carcinoma by HADHA-mediated lipid reprogramming. J Hematol Oncol. (2020) 13:12. doi: 10.1186/s13045-019-0841-3, PMID: 32033570 PMC7006096

[46] Fang H Li H Zhang H Wang S Xu S Chang L . Short-chain L-3-hydroxyacyl-CoA dehydrogenase: A novel vital oncogene or tumor suppressor gene in cancers. Front Pharmacol. (2022) 13:1019312. doi: 10.3389/fphar.2022.1019312, PMID: 36313354 PMC9614034

[47] Jiang M Cui H Liu Z Zhou X Zhang L Cao L . The Role of Amino Acid Metabolism of Tumor Associated Macrophages in the Development of Colorectal Cancer. Cells. (2022) 11:4106. doi: 10.3390/cells11244106, PMID: 36552870 PMC9776905

[48] Lian S Liu S Wu A Yin L Li L Zeng L . Branched-Chain Amino Acid Degradation Pathway was Inactivated in Colorectal Cancer: Results from a Proteomics Study. J Cancer. (2024) 15:3724–37. doi: 10.7150/jca.95454, PMID: 38911385 PMC11190764

[49] Ren J Feng J Song W Wang C Ge Y Fu T . Development and validation of a metabolic gene signature for predicting overall survival in patients with colon cancer. Clin Exp Med. (2020) 20:535–44. doi: 10.1007/s10238-020-00652-1, PMID: 32772211

[50] Nwosu ZC Battello N Rothley M Piorońska W Sitek B Ebert MP . Liver cancer cell lines distinctly mimic the metabolic gene expression pattern of the corresponding human tumours. J Exp Clin Cancer Res. (2018) 37:211. doi: 10.1186/s13046-018-0872-6, PMID: 30176945 PMC6122702

[51] Wang X Song H Liang J Jia Y Zhang Y . Abnormal expression of HADH, an enzyme of fatty acid oxidation, affects tumor development and prognosis (Review). Mol Med Rep. (2022) 26:355. doi: 10.3892/mmr.2022.12871, PMID: 36239258 PMC9607826

[52] Chu C Liu S He Z Wu M Xia J Zeng H . HADH suppresses clear cell renal cell carcinoma progression through reduced NRF2-dependent glutathione synthesis. Transl Oncol. (2024) 49:102112. doi: 10.1016/j.tranon.2024.102112, PMID: 39226735 PMC11402447

