# Supplementary material for: Liquid-liquid phase separation-driven molecular subtyping and prognostic modeling in colorectal cancer
Source: Front Immunol. 2026 Jan 9;16:1741979. doi: 10.3389/fimmu.2025.1741979 (PMC12827789; doi:10.3389/fimmu.2025.1741979)
Supplement: Supplementary Figure 1 — Spatial transcriptomics analysis of the CRC pathological section. (A) The hematoxylin-eosin staining of the pathologic section. (B) The spatial localization of all cell types. Each dot represented a microregion, and different colors represented different cell types. (C) Distribution of malignant and nonmalignant regions. (D) AUC scores of modules containing 430 LLPS-related genes. (E) Correlation between AUC scores and different cell types. [file Supplementaryfile1.docx]

**Supplemental materials**

Inventory of supplementary materials

1. Table S1………….…………………Page 2
2. Table S3………….…………………Page 3
3. Table S4………….…………………Page 4
4. Figure S1………….………………. Page 5
5. Figure S2………….………………. Page 6
6. Figure S3………….………………. Page 7
7. Figure S4………….………………. Page 8
8. Figure S5………….………………. Page 9
9. Figure S6………….………………. Page 10
10. Figure S7………….………………. Page 11

| Dataset | GSE39582 | GSE17536 | COAD | READ |
| --- | --- | --- | --- | --- |
| Database | GEO | GEO | TCGA | TCGA |
| Number of tumor samples | 566 | 177 | 430 | 154 |
| Age (mean ± SD; years) | 66.906 ± 13.267 | 65.48 ± 13.082 | 66.44 ± 12.793 | 64.62 ± 11.232 |
| Gender |  |  |  |  |
| Male | 310 (54.8%) | 96 (54.2%) | 232 (54.0%) | 86 (55.8%) |
| Female | 256 (45.2%) | 81 (45.8%) | 198 (46.0%) | 68 (44.2%) |
| Survival status |  |  |  |  |
| Alive | 371 (65.5%) | 104 (58.8%) | 336 (78.1%) | 127 (82.5%) |
| Dead | 191 (33.7%) | 73 (41.2%) | 94 (21.9%) | 27 (17.5%) |
| NA | 4 (0.8%) | 0 | 0 | 0 |
| Median survival time (months) | 51 | 42.27 | 22.38 | 21.23 |
| T classification |  |  |  |  |
| 1 | 11 (1.9%) | / | 12 (2.8%) | 8 (5.2%) |
| 2 | 45 (8.0%) | / | 75 (17.4%) | 27 (17.5%) |
| 3 | 367 (64.8%) | / | 294 (68.4%) | 105 (68.2%) |
| 4 | 119 (21.0%) | / | 49 (11.4%) | 13 (8.4%) |
| NA | 24 (4.3%) | / | 0 | 1 (0.7%) |
| N classification |  |  |  |  |
| 0 | 302 (53.4%) | / | 253 (58.8%) | 78 (50.6%) |
| 1 | 134 (23.7%) | / | 100 (23.3%) | 42 (27.3%) |
| 2 | 104 (18.4%) | / | 77 (17.9%) | 31 (20.1%) |
| NA | 26 (4.5%) | / | 0 | 3 (2.0%) |
| M classification |  |  |  |  |
| 0 | 482 (85.2%) | / | 318 (74.0%) | 117 (76.0%) |
| 1 | 61 (10.8%) | / | 60 (14.0%) | 22 (14.3%) |
| NA | 23 (4.0%) | / | 52 (12.0%) | 15 (9.7%) |
| Clinical stage |  |  |  |  |
| I | 33 (5.8%) | 24 (13.6%) | 73 (17.0%) | 28 (18.2%) |
| II | 264 (46.6%) | 57 (32.2%) | 165 (38.4%) | 47 (30.5%) |
| III | 205 (36.2%) | 57 (32.2%) | 121 (28.1%) | 47 (30.5%) |
| IV | 60 (10.6%) | 39 (22.0%) | 60 (14.0%) | 23 (14.9%) |
| NA | 4 (0.8%) | 0 | 11 (2.5%) | 9 (5.9%) |

**Table S1** Details of the bulk RNA sequencing datasets from the study.

## Table S3 Sequences of the primers used for PCR.

| Gene | Species |  | Sequence |
| --- | --- | --- | --- |
| AQP11 | Homo | Forward | CACTTCCAGGAAGTCCGAACCA |
|  |  | Reverse | GTAGCGAAAGTGCCAAAGCTGG |
| CCDC34 | Homo | Forward | AAAGTGGCGAGCCTGAGAGGAA |
|  |  | Reverse | ATGGTGTCAGGCGGCTTTCTGG |
| FBL | Homo | Forward | GAGGCTTCCATTCTGGTGGCAA |
|  |  | Reverse | CAGGTTCTTGGTGACCAGTGCA |
| HADH | Homo | Forward | TCCGTTGTCCACAGCACAGACT |
|  |  | Reverse | GGAGGAAGTGTTGCTGGCAAAG |
| RAB15 | Homo | Forward | CTTCACCGACAACGAGTTCCAC |
|  |  | Reverse | GATGGTCTGGTATCTCTCCTGC |
| β-actin | Homo | Forward | GAAGAGCTACGAGCTGCCTGA |
|  |  | Reverse | CAGACAGCACTGTGTTGGCG |

**Table S4** Characteristics between high and low HADH expression

subgroups in CRC patients.

| Characteristics | HADH low expression | HADH high expression | P value |
| --- | --- | --- | --- |
| Number | 42 | 38 |  |
| **IRS score** | 2 ± 0.765 | 5.50 ± 1.409 | **＜0.001***** |
| Age | 60.00 ± 16.612 | 61.01 ± 12.463 | 0.719 |
| Gender |  |  | 0.621 |
| Male | 22 (52.4%) | 22 (57.9%) |  |
| Female | 20 (47.6%) | 16 (42.1%) |  |
| **Grade** |  |  | **＜0.001***** |
| 1 | 0 | 5 (13.2%) |  |
| 2 | 28 (66.7%) | 32 (84.2%) |  |
| 3 | 14 (33.3%) | 1 (2.6%) |  |
| Location |  |  | 0.224 |
| Left | 20 (47.6%) | 13 (34.2%) |  |
| Right | 22 (52.4%) | 25 (65.8%) |  |
| MMR status |  |  | 0.616 |
| dMMR | 7 (16.7%) | 8 (21.1%) |  |
| pMMR | 35 (83.3%) | 30 (78.9%) |  |
| Tumor size | 6.49 ± 2.935 | 5.95 ± 2.975 | 0.416 |
| Survival status |  |  | 0.595 |
| Alive | 31 (73.8%) | 26 (68.4%) |  |
| Dead | 11 (26.2%) | 12 (31.6%) |  |
| T classification |  |  | 0.601 |
| T1-2 | 3 (7.1%) | 5 (13.2%) |  |
| T3-4 | 39 (92.9%) | 33 (86.8%) |  |
| N classification |  |  | 0.224 |
| N0 | 22 (52.4%) | 25 (65.8%) |  |
| N1-2 | 20 (47.6%) | 13 (34.2%) |  |
| M classification |  |  | 0.090 |
| M0 | 33 (78.6%) | 35 (92.1%) |  |
| M1 | 9 (19.0%) | 3 (7.9%) |  |
| **TNM stage** |  |  | **0.045*** |
| Stage I-II | 16 (38.1%) | 23 (60.5%) |  |
| Stage III-IV | 26 (61.9%) | 15 (39.5%) |  |


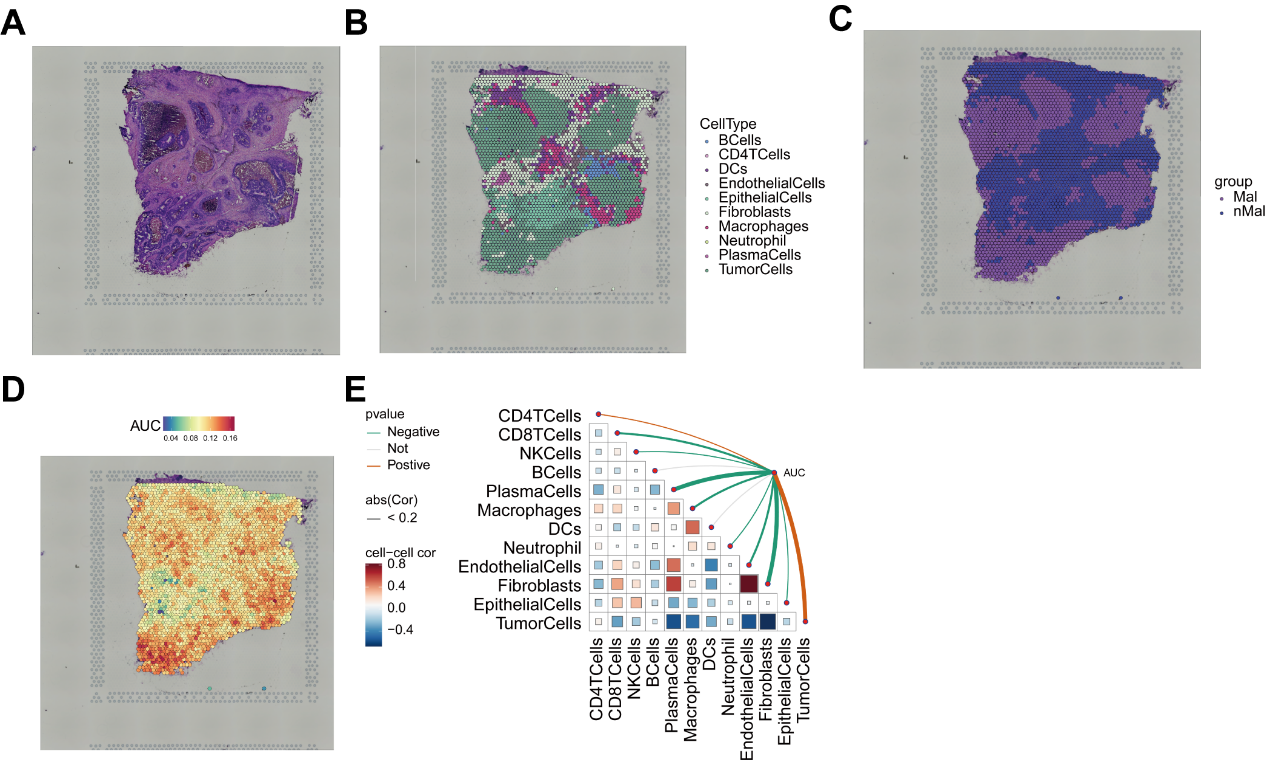


Figure S1 Spatial transcriptomics analysis of the CRC pathological section. (A) The hematoxylin-eosin staining of the pathologic section. (B) The spatial localization of all cell types. Each dot represented a microregion, and different colors represented different cell types. (C) Distribution of malignant and nonmalignant regions. (D) AUC scores of modules containing 430 LLPS-related genes. (E) Correlation between AUC scores and different cell types.


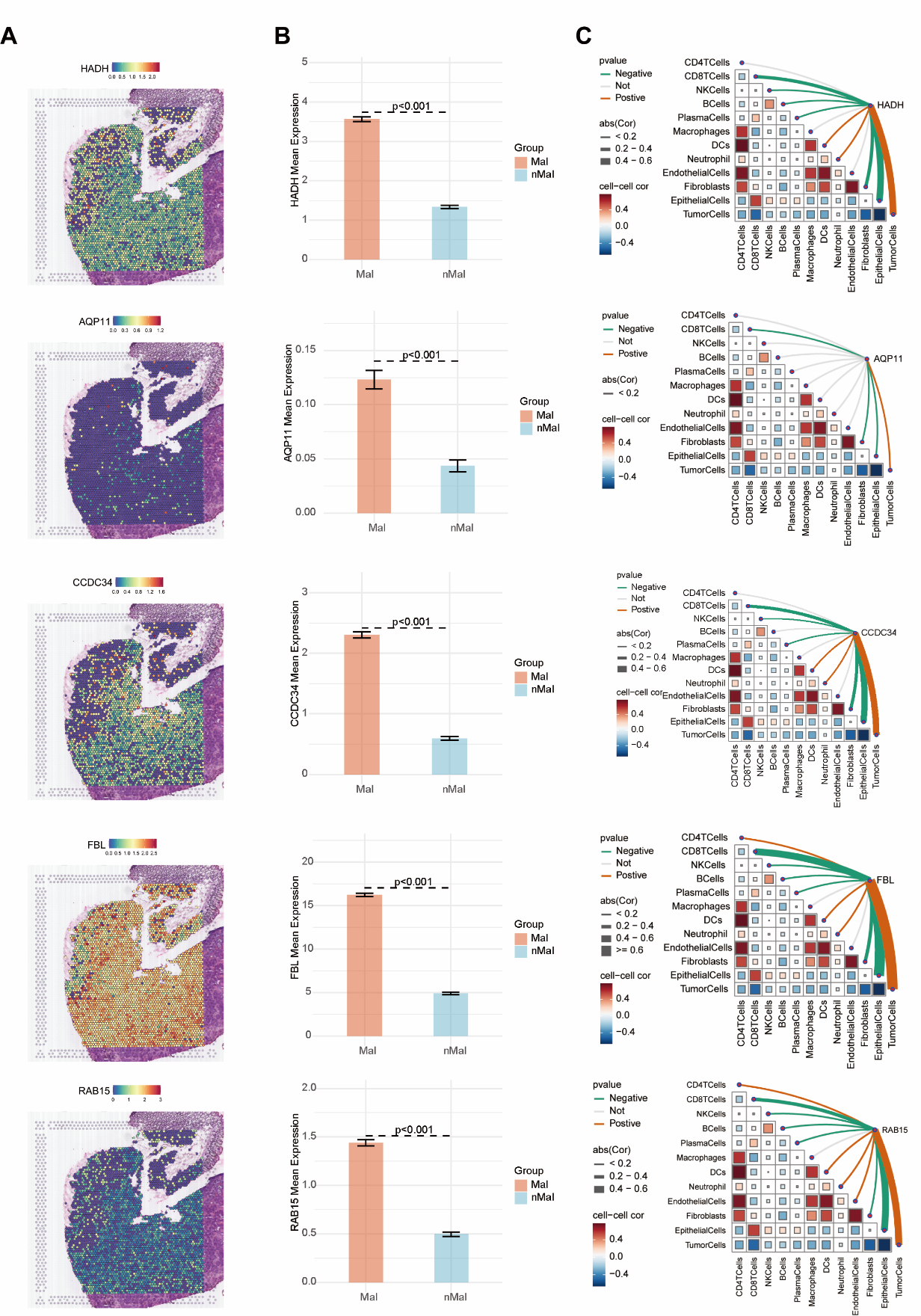


Figure S2 Spatial transcriptomics analysis of the second CRC pathological section. (A) The spatial localization of the five genes in each microregion. (B) The expression levels of the five genes in the malignant and nonmalignant groups. (C) The correlation between the five genes and different cell types.


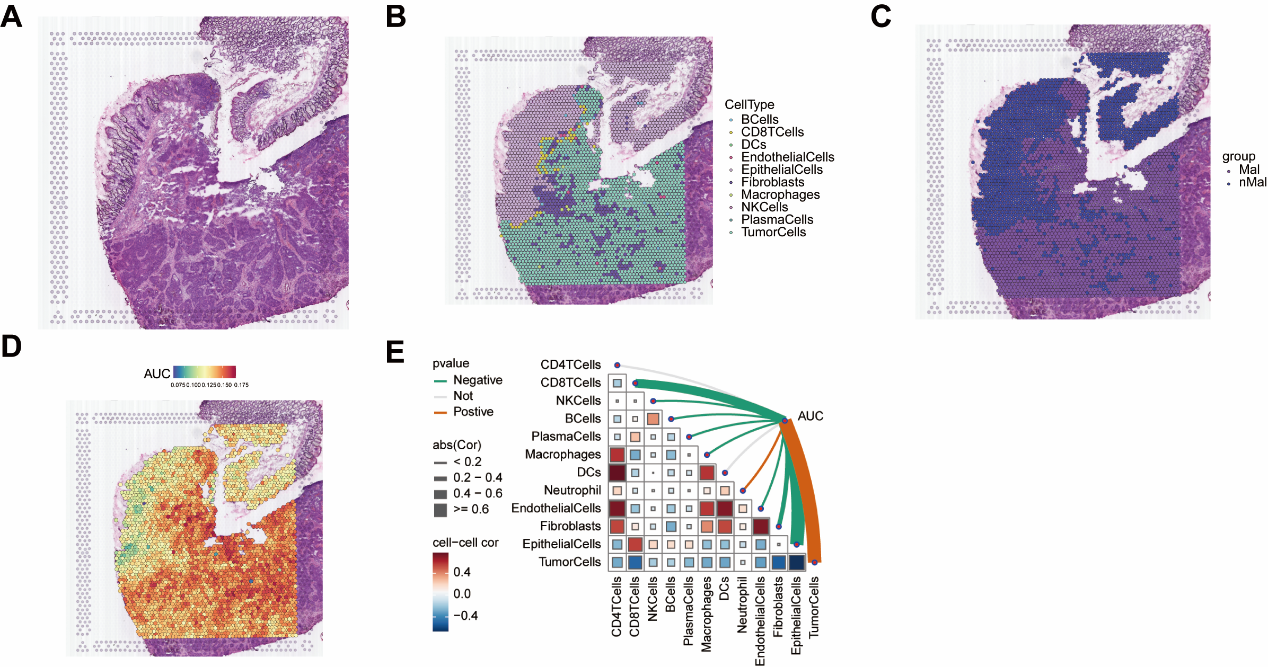


Figure S3 Spatial transcriptomics analysis of the second CRC pathological section. (A) The hematoxylin-eosin staining of the pathologic section. (B) The spatial localization of all cell types. Each dot represented a microregion, and different colors represented different cell types. (C) Distribution of malignant and nonmalignant regions. (D) AUC scores of modules containing 430 LLPS-related genes. (E) Correlation between AUC scores and different cell types.


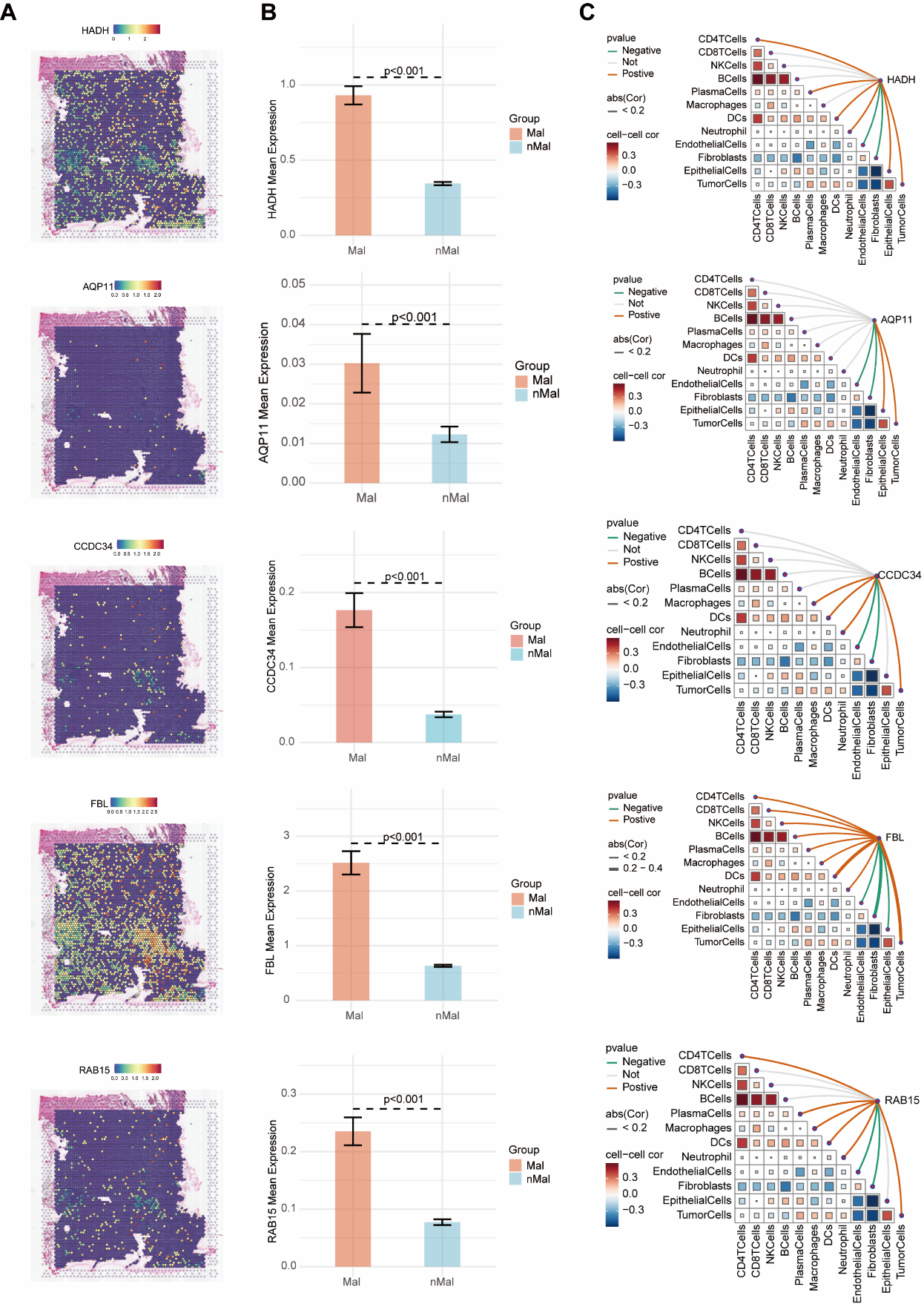


Figure S4 Spatial transcriptomics analysis of the third CRC pathological section. (A) The spatial localization of the five genes in each microregion. (B) The expression levels of the five genes in the malignant and nonmalignant groups. (C) The correlation between the five genes and different cell types.


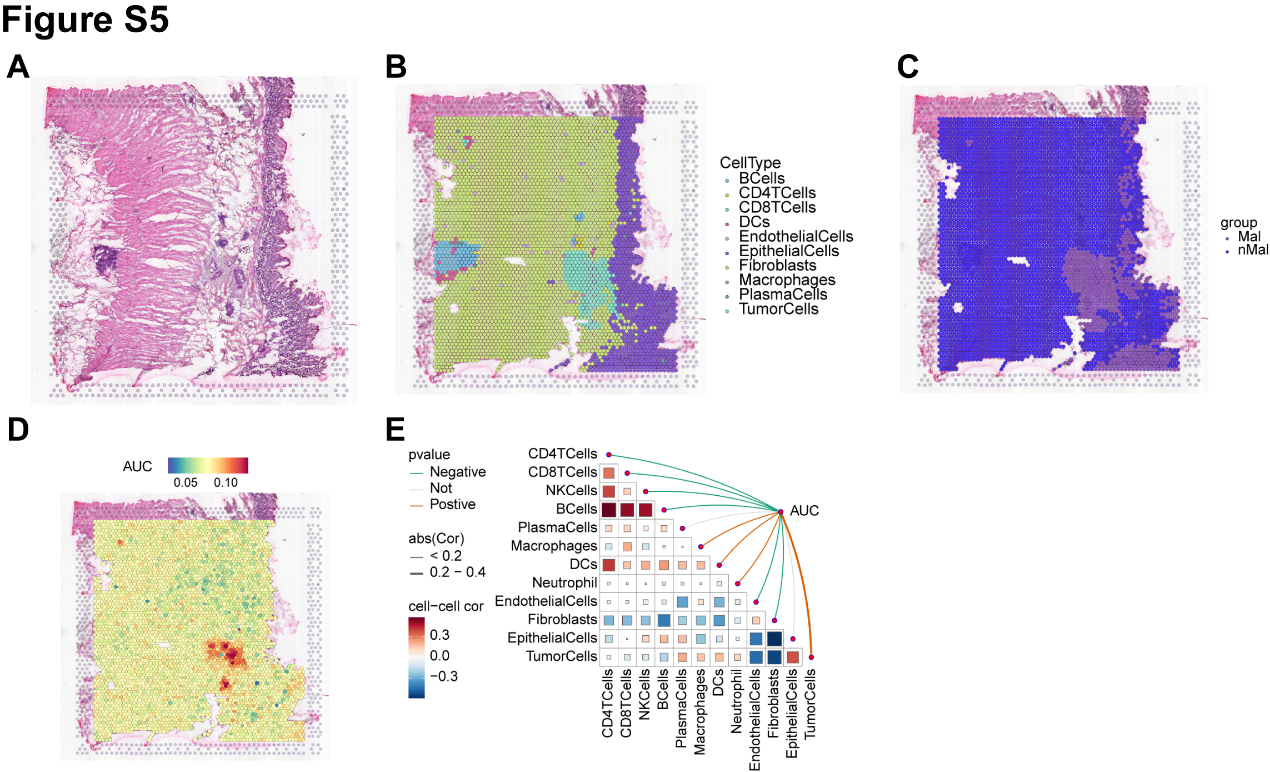


Figure S5 Spatial transcriptomics analysis of the third CRC pathological section. (A) The hematoxylin-eosin staining of the pathologic section. (B) The spatial localization of all cell types. Each dot represented a microregion, and different colors represented different cell types. (C) Distribution of malignant and nonmalignant regions. (D) AUC scores of modules containing 430 LLPS-related genes. (E) Correlation between AUC scores and different cell types.


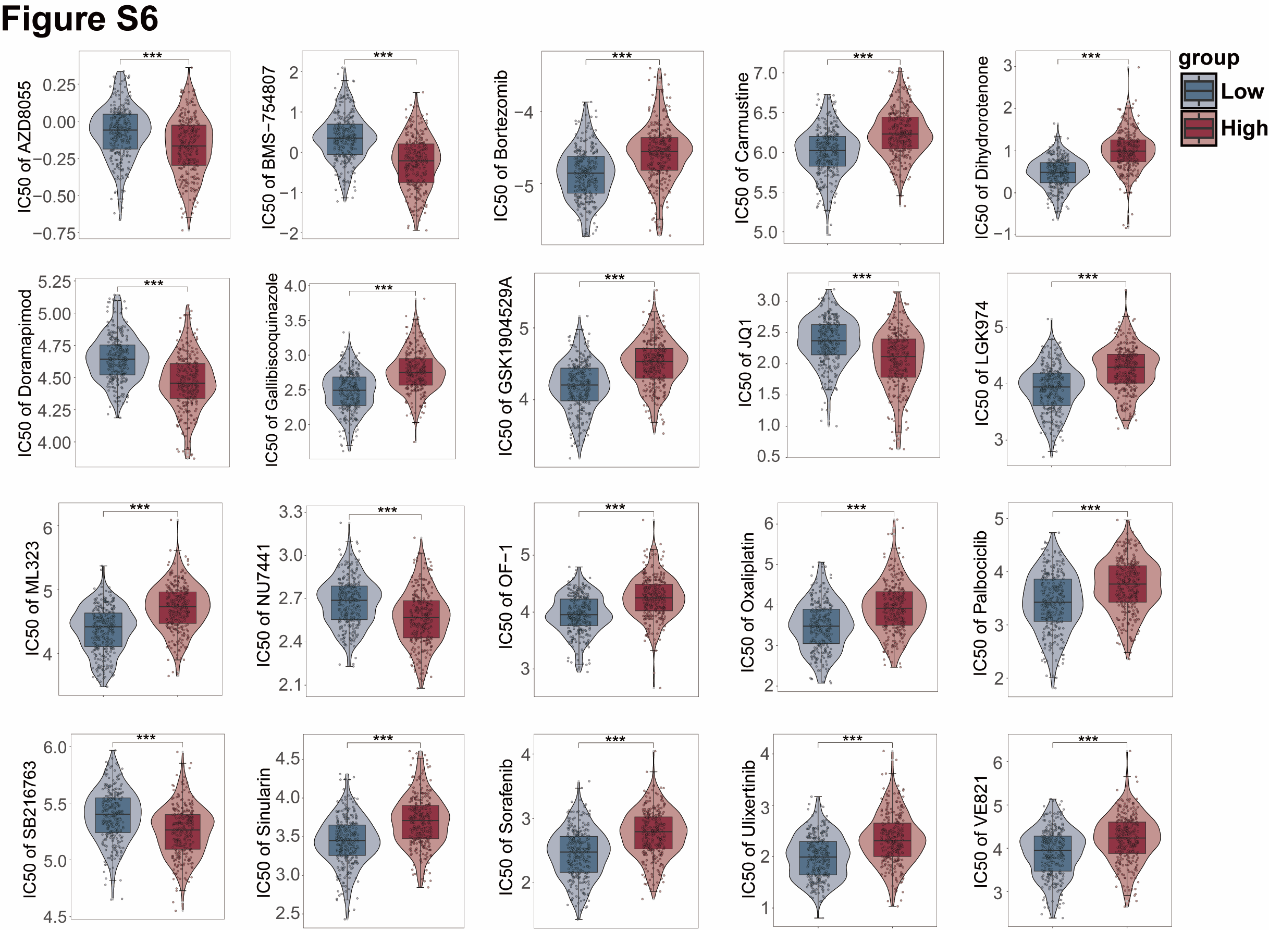


Figure S6 Comparisons of IC50 of twenty drugs in the high- and low-risk groups. *** p<0.001.


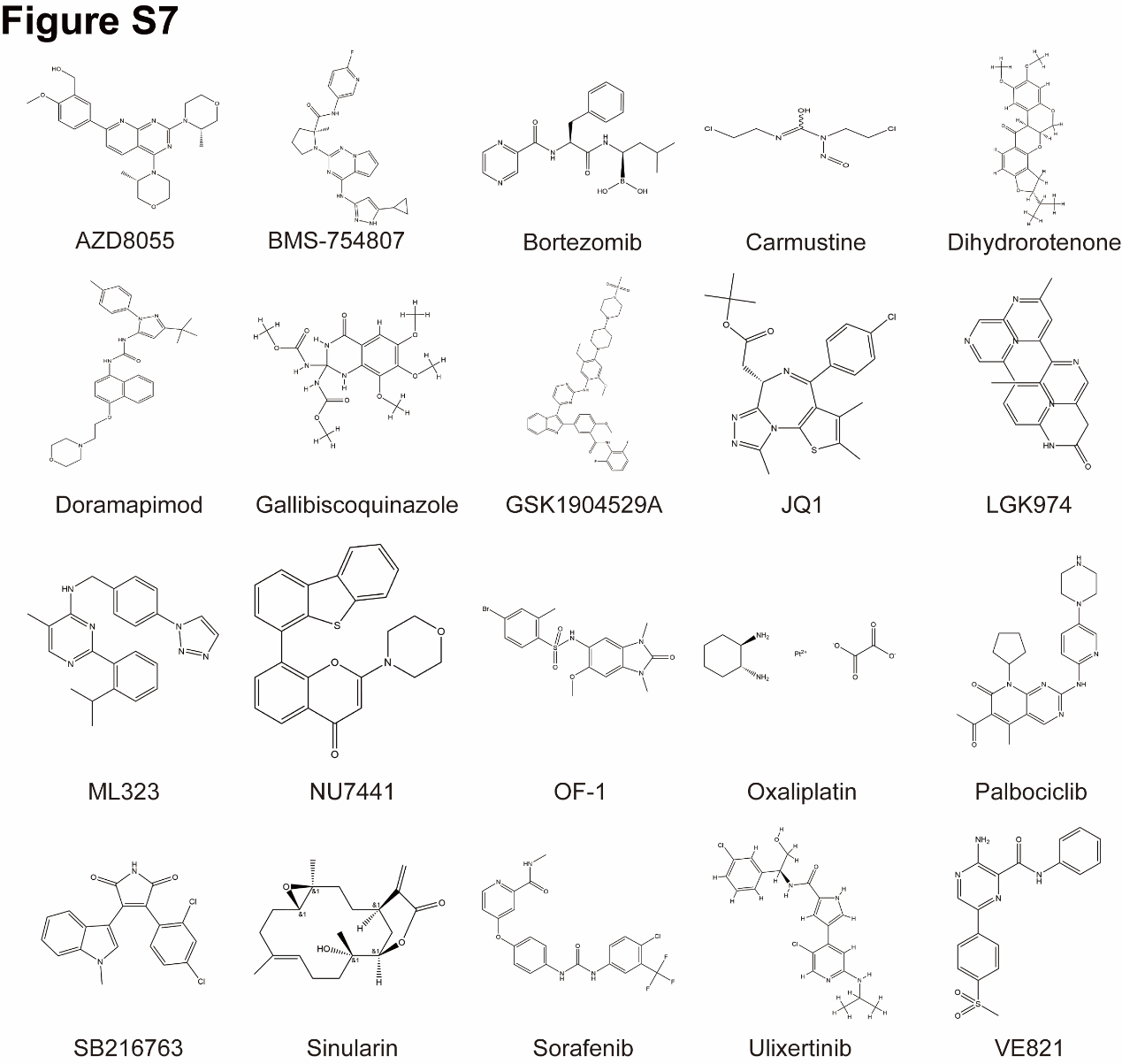


Figure S7 Chemical structures of twenty drugs.
